# Beat-to-Beat Patterning of Sinus Rhythm Reveals Non-linear Rhythm in the Dog Compared to the Human

**DOI:** 10.3389/fphys.2019.01548

**Published:** 2020-01-22

**Authors:** N. Sydney Moïse, Wyatt H. Flanders, Romain Pariaut

**Affiliations:** ^1^College of Veterinary Medicine, Department of Clinical Sciences, Cornell University, Ithaca, NY, United States; ^2^Department of Physics, University of Washington, Seattle, WA, United States

**Keywords:** sinus arrhythmia, heart rate variability, Poincaré plots, Holter monitoring, parasympathetic, sinus node, non-linear, autonomic nervous system

## Abstract

The human and dog have sinus arrhythmia; however, the beat-to-beat interval changes were hypothesized to be different. Geometric analyses (R–R interval tachograms, dynamic Poincaré plots) to examine rate changes on a beat-to-beat basis were analyzed along with time and frequency domain heart rate variability from 40 human and 130 canine 24-h electrocardiographic recordings. Humans had bell-shaped beat-interval distributions, narrow interval bands across time with continuous interval change and linear changes in rate. In contrast, dogs had skewed non-singular beat distributions, wide interval bands {despite faster average heart rate of dogs [mean (range); 81 (64–119)] bpm compared to humans [74.5 (59–103) *p* = 0.005]} with regions displaying a paucity of intervals (*zone of avoidance)* and linear plus non-linear rate changes. In the dog, dynamic Poincaré plots showed linear rate changes as intervals prolonged until a point of divergence from the line of identity at a mean interval of 598.5 (95% CI: 583.5–613.5) ms (*bifurcation interval*). The dog had bimodal beat distribution during sleep with slower rates and greater variability than during active hours that showed singular interval distributions, higher rates and less variability. During sleep, Poincaré plots of the dog had clustered or branched patterns of intervals. A slower rate supported greater parasympathetic modulation with a branched compared to the clustered distribution. Treatment with atropine eliminated the non-linear patterns, while hydromorphone shifted the bifurcated branching and beat clustering to longer intervals. These results demonstrate the unique non-linear nature of beat-to-beat variability in the dog compared to humans with increases in interval duration (decrease heart rate). These results provoke the possibility that changes are linear with a dominant sympathetic modulation and non-linear with a dominant parasympathetic modulation. The abrupt bifurcation, zone of avoidance and beat-to-beat patterning are concordant with other studies demonstrating the development of exit block from the sinus node with parasympathetic modulation influencing not only the oscillation of the pacing cells, but conduction to the atria. Studies are required to associate the *in vivo* sinus node beat patterns identified in this study to the mapping of sinus impulse origin and exit from the sinus node.

## Introduction

Arising from the normal sinus node the relationship of each beat to that of the next is dependent on the intrinsic characteristics of the pacing cells (Gao et al., 2010; Nicolini et al., 2012; Monfredi et al., 2013; Yaniv et al., 2014a, b; Valente et al., 2018) and the external forces of the autonomic nervous system (Billman, 2011). Recent studies reveal that the stochastic (unpredictable) (Yaniv et al., 2014b) and chaotic (deterministic) (Nicolini et al., 2012; Yaniv et al., 2014b; Zhang et al., 2015) mechanisms that regulate the beating heart are caused by the complexity of structure (Fedorov et al., 2009; Ambrosi et al., 2012) and function of the sinus node in both the dog and human (Fedorov et al., 2009, 2010, 2012; Glukhov et al., 2013; Csepe et al., 2016; Kalyanasundaram et al., 2019). The sinus node is not simply a cluster of the most rapidly depolarizing cells of the heart. Instead, it is a central core surrounded by a transitional region of specialized cells, fibrous tissue, and atrial myocytes. After spontaneous depolarization a sinus impulse traverses specialized conduction pathways to depolarize the atria (Kalyanasundaram et al., 2019). The rhythm so recognized in the dog of sinus arrhythmia results from dynamic inputs of sympathetic and parasympathetic systems triggered by a combination of central medullary influences, cardiovascular reflexes, and mechanics of respiration (Boineau et al., 1980; Goldberger et al., 1994; Roossien et al., 1997; Brodde et al., 1998; Yasuma and Hayano, 2004; Billman, 2011, 2013; Krohova et al., 2018).

Long-term electrocardiographic (Holter) recordings have permitted the appraisal of rhythm during variation of autonomic input throughout the day. Time, (Malik et al., 1996; Shaffer and Ginsberg, 2017) frequency, (Csepe et al., 2016) and geometric (tachograms, histograms, Poincaré plots) (Esperer et al., 2008; Khandoker et al., 2013; Borracci et al., 2018) domain indices are often used in the assessment of heart rate (sinus) variability (Malik et al., 1996; Billman, 2011; Billman et al., 2015b; Shaffer and Ginsberg, 2017). Furthermore, to understand better the complexity of biophysical oscillators like the sinus node, advanced methods have been developed to analyze heart rate variability such as measures of fractal-like behavior (long-term analysis), detrended fluctuation (Stein et al., 2005) (short-term analysis), disorder (approximate and sample entropy), non-linear dynamical systems (multiscale Poincaré plots) (Henriques et al., 2015; Shaffer and Ginsberg, 2017) and chaotic behavior (Nicolini et al., 2012; Sassi et al., 2015; Zhang et al., 2015; Behar et al., 2018b; Valente et al., 2018).

The dog and human exhibit sinus arrhythmia; however, the variation between beat intervals is greater in the dog (Behar et al., 2018a, b; Kalyanasundaram et al., 2019). The complexity and mechanism for the dramatic variation in the beat intervals so unique to the dog is explained inadequately. The explanation of high parasympathetic tone does not elucidate the underlying mechanism for the variation, nor does it explain the different patterns of sinus arrhythmia commonly identified on the canine electrocardiogram. The integration of G-protein coupled receptors that with activation reduce the heart rate and regulators of G-protein signaling that attenuate the parasympathetic signaling acting through M2 muscarinic and adenosine receptors that control ion channels responsible for the autorhythmic depolarization of the sinus node cells (Mighiu and Heximer, 2012) is key in the dog with normal or abnormal sinus node function.

We, and others, have observed the unique beat patterning of changes in heart rate in the dog using geometric heart rate variability (Moïse et al., 2010; Gladuli et al., 2011; Blake et al., 2018). However, as the heart rhythm is non-stationary and two-dimensional methods do not permit thorough examination, expanded techniques are required to understand the dynamics of heart rate variability. In the observational portion of the study herein, we used new methodologies (three-dimensional tachograms, dynamic Poincaré plots, three-dimensional histographic Poincaré plots) to glean new insights into our understanding of sinus arrhythmia in the dog as it compares to the human. Our observations led to the following hypotheses. We hypothesized that our new methods to examine beat-to-beat dynamics during sinus arrhythmia would reveal a unique non-linear beat patterning in the dog that differed from humans. We hypothesized that particular beat patterning of sinus arrhythmia is associated with different levels of parasympathetic input as indirectly reflected by indices of heart rate variability and through studies in the dog whereby parasympatholytic and parasympathomimetic drugs altered the beat-to-beat patterns. We further hypothesized that the rate at which the beat-to-beat interval in the dog deviates from a linear slowing (bifurcation interval) approximates the intrinsic rate of oscillation of the canine sinus node. Finally, from our data on 130 dogs and 40 humans with clinically normal sinus node function, we propose hypotheses for future studies concerning the mechanistic difference in the patterning of sinus arrhythmia between these two species.

## Materials and Methods

### Human Holter Recording Database

The 24-h ambulatory ECG recordings (Holter recordings) from 200 healthy adult humans were accessed from the Telemetric and Holter ECG Warehouse (THEW) maintained by the University of Rochester Medical Center, Rochester, NY, United States. Permission to use these data was approved by the review board after submission of proposal for use via http://www.thew-project.org/. Reasons for performing the Holter recordings were not given for this database. The enrollment criteria for selection from this data set included the following: no overt cardiovascular disease, no history of cardiovascular disease, no systemic hypertension or chronic illnesses, adults > 18-years of age, normal physical examination, no medications that would interfere with sinus rate, normal echocardiographic examination, no pregnancy, no diagnosis of sinus node dysfunction, no sinus pauses > 2500 ms, < 3.0% or < 4000 ventricular ectopic complexes, < 0.25% atrial ectopic complexes and no abnormal symptoms during Holter recording. It is noted that the only difference in the criteria between the human and dog concerns the duration of sinus pauses. None of the humans had pause durations > 2500 ms. However, this is a common finding in the dog. The goal of these criteria was not to select recordings devoid of any abnormalities, but to select those for which sinus node function was deemed normal and the presence of arrhythmias not of clinical importance.

### Canine Holter Recording Database

Holter recordings of dogs were retrieved from the database of the Section of Cardiology Holter Laboratory at the College of Veterinary Medicine, Cornell University, Ithaca, NY, United States (analyzing canine recordings since 1988). A database of > 5000 Holter recordings between 2009 and 2014 was examined to identify only those performed using Forest Medical Holter recorders (Trillium 5000/5900) (Syracuse, NY, United States). Each recording was required to have a minimum of 22 h with 99% artifact free data. All recordings were from clinical patients. Recordings in these dogs were performed to screen for arrhythmias, to determine if suspected arrhythmias identified by either auscultation or during electrocardiographic monitoring were of clinical importance, or to investigate cause of syncope or seizure. Recordings were selected only if sinus node function was determined to be normal. The enrollment criteria for this data set included the following: no overt cardiovascular disease (including myocardial failure or congestive heart failure), no systemic diseases, adult dog > 1-year of age, no physical abnormalities that would affect sinus rate, no medications that would influence sinus rate, no pregnancy, no diagnosis of sinus node dysfunction, no sinus pauses > 5500 ms, no more than 3 pauses > 4000 ms, < 3.0% or < 4000 ventricular ectopic complexes, < 0.25% atrial ectopic complexes and no abnormal clinical signs during Holter recording. Additionally, any Holters that had evidence of vasovagal reflex or Bezold-Jarisch reflex in association with a history of syncope were excluded. Therefore, the goal of these criteria was not to select recordings devoid of any abnormalities (so that aged dogs could be included), but to select those for which sinus node function was deemed normal and the presence of arrhythmias not of clinical importance. Clinical sinus node dysfunction in the dog is characterized by an average heart rate < 60 bpm, minimum heart rate < 30 bpm, time with heart rate < 50 bpm > 350 min, number of pauses > 2 s > 1500, longest sinus pause > 5500 ms and > 3 pauses > 4 s. All 24-h electrocardiographic recordings were performed with the dogs in the home environment.

Owners were given a detailed diary form to complete so that sleep-wake cycles could be documented. The Forest Medical Holter recorders (Trillium^®^ 5000/5900) provide 256 Hz sampling frequency signals (4 ms time resolution) with 8-bit amplitude resolution (5 μV amplitude resolution). Leads were positioned for a 3-lead modified orthogonal X, Y, Z configuration. After downloading the raw data, a technician trained in cardiac rhythm and Holter analysis edited the recordings. All recordings were then over-read by a veterinary cardiologist (NSM) to ensure > 99% accuracy for the identification and classification of P and QRS waves given the importance for heart rate variability analysis (Peltola, 2012). It is emphasized that a veterinary cardiologist with extensive experience in the analysis of canine electrocardiographic recordings reviewed all electrocardiograms to ensure that identified complexes were sinus in origin and not atrial premature complexes. Others unfamiliar with the rhythms of the dog mistakenly identify normal sinus beats as atrial premature complexes because of the prominent sinus arrhythmia. To implement these analyses, software from Forest Medical (Trillium^®^), was used for the QRS detection, beat annotations, time domain and frequency domain heart rate variability. Additionally, Forest Medical permitted access to the raw data (R–R intervals and annotations) for the development of additional software (WHF).

### Sinus Rate Determination

For both the human and canine recordings, the files were examined to ensure that the R–R intervals represented the P–P intervals. Throughout this manuscript the term R–R interval will be used as a surrogate for P–P interval. When referring to the relationship between an R–R interval and the next, the term beat-to-beat interval will be used. Any recordings with atrioventricular conduction block, QRS complexes not preceded by a P wave (e.g., junctional origin) or any other arrhythmias that would be annotated as a normal complex, were not included in the analyses for the assessment of sinus node rate and rhythm. Descriptive data for sinus rate were determined including the average, minimum and maximum rate, time with rate < 50 bpm, time with rate > 120 bpm, number of pauses > 2 s and the longest pause. The density of specific R–R intervals was shown as histograms for each hour and summed for the full 24-h. Additionally, heart rate over time was graphically displayed across time with heart rate tachograms and two- and three-dimensional R–R interval tachograms. On the heart rate tachogram the rolling eight-beat average rate was shown between the minimum and maximum rate for each segment. The two-dimensional tachogram plotted the R–R interval for each hour and over 24-h. Importantly, because each recording contained approximately 100,000 data points, an overlay of points did not permit an appreciation of interval density (number of intervals with same or similar values inadequately identified) and this prompted the development of customized software (WHF).

### Heart Rate Variability Analyses

Based on the guidelines for heart rate variability analysis time, (Malik et al., 1996; Shaffer and Ginsberg, 2017) frequency, and geometric domain analyses were performed (Trillium^®^). Time domain methods included (1) estimate of overall heart rate variability using standard deviation of all beats (SDNN), (2) estimate of long-term components of heart rate variability using standard deviation of all 5-min beat interval means (SDANN) and (3) cycles shorter than 5 min were assessed by the mean of all 5-min beat interval standard deviations (SDNNIn), (4) estimate of short-term components of heart rate variability using the square root of the mean squared successive interval differences (RMSSD). The frequency domain parameters analyzed were total power and high frequency. For the frequency domain analyses a window of 512 beats with a Hamming filter applied was used. Total power density was determined with frequencies < 0.4 Hz and high frequency was in the range of 0.15–0.4 Hz. During the hour-windows of the frequency analysis, the 512 beat window was initially selected at the midpoint in the hour so long as the average heart rate approximated that of the average for that hour ± 5 bpm. The window was moved from the midpoint before or after until this heart rate was found. During sleep hours with stable rate and rhythm, time and frequency domain parameters were corrected for heart rate. To correct for the mathematical influence of rate on variability, time domain indices were divided by the average interval of the examined period and frequency domain indices were divided by the square of the average interval in seconds (Sacha and Pluta, 2008; Billman, 2013; Sacha et al., 2013; Billman et al., 2015b).

### Beat-to-Beat and Three-Dimensional Analyses

All Holter recordings were examined using geometric domain heart rate variability to identify beat-to-beat patterns. Two-dimensional Poincaré plots were constructed for each hour and 24-h by plotting an R–R interval on the x-axis and the next interval (R–R + 1 interval) on the y-axis. In order to examine the beat-to-beat patterning it was necessary to create the ability to select periods for analysis such as hours of activity or stable sleep hour, while also having the ability to visualize the formation of the Poincaré plots in two and three-dimensions. Therefore, advanced geometric domain analyses including dynamic Poincaré plots, three-dimensional histographic Poincaré and three-dimensional R–R interval tachograms were developed by one of us (WHF) using JavaScript HTML and GLSL^1^ ^,2^, after integration with the time series R–R intervals. Three-dimensional histographic Poincaré plots were generated by sorting the interval data and logarithmically adding onto a GPU based on different regions. A user interface was developed for a web-based amalgamation to permit interval and time selection. The dynamic Poincaré plots were used to specifically follow the patterns during acceleration and slowing of the heart rate. Examination of these plots, in addition to the R–R interval tachograms, prompted the development of pattern classification that then led to further analyses and comparisons.

(1) Regions with a paucity (also coined as a ‘zone of avoidance’) (Moïse et al., 2010) of R–R intervals seen on the tachograms and beat-to-beat intervals seen on the Poincaré plots.
(2) Regions with clustered beat-to-beat intervals (defined as discontinuous and isolated intervals on the Poincaré plots coupled with dense banding separated by a paucity of R–R intervals on the tachogram and bimodal distribution on histograms) (13 boxers/12 non-boxers).
(3) Regions with branched beat-to-beat intervals (defined as continuous intervals on the Poincaré plots coupled with broad spreading of R–R intervals on the tachogram and rightward-skewed distribution on histograms) (13 boxers/12 non-boxers).
(4) R–R interval bifurcation (this interval determined as the average of the three shortest intervals during a stable sleep hour in 25 boxers and 25 non-boxers).

### Parasympatholytic and Parasympathomimetic Drug Effects

The findings of beat patterning during spontaneous sinus rhythm in the dog strongly suggested the influence of the parasympathetic nervous system. After approval from the Institutional Animal Care and Use Committee, 8 beagles had 24-h electrocardiographic recordings to assess the geometric beat-to-beat patterning as influenced by parasympatholytic and parasympathomimetic agents. Atropine (0.04 mg/kg intravenously) and hydromorphone (0.2 mg/kg intravenously) were administered on separate days after a baseline recording was obtained. Regions of the recordings associated with these treatments were analyzed for beat-to-beat patterns. The corresponding time of the baseline recording served as the control period.

### Statistical Analysis

The following comparisons were made (1) 24-h data for boxer dog breed, non-boxer breeds and human (2) hour for all dogs with bimodal versus singular interval density, and (3) stable sleep hour with clustered versus branched interval distribution in the dogs. Additionally, the bifurcation interval in the dog was examined relative to age, average heart rate and time domain indices of heart rate variability SDNN (the latter after correction for heart rate). Distribution of all continuous variables was assessed for normality. Difference between groups for variables with normal distribution were analyzed using a *t*-test and data is presented as mean and standard deviation. Differences between those variables showing a non-normal distribution were analyzed with non-parametric methods (Wilcoxon Rank Sum test, also called Mann–Whitney-Test) and data is presented as median and range. Adjustments for *p*-values controlling for multiple comparisons was done using Bonferroni correction. The relationship between variables of heart rate and heart rate variability with age for each group (boxers, non-boxers, and humans) was examined by a regression analysis with slopes and standard error reported. Differences in the time domain parameters between these groups was tested with a one-way ANOVA followed with a *post hoc* multiple comparisons with Tukey correction. Differences between groups for categorical variables were tested using Fisher’s exact test. Differences for paired data (singular/bimodal distribution) within a category (boxer, non-boxer) were achieved with paired data non-parametric Wilcoxon Signed Rank test. Correlation between continuous variables was explored by using Pearson correlation for parametric data and Spearman for non-parametric. To determine relationship bivariate analysis was performed for specific variables. All analyses were performed in JMP (v.12.0.1 and v. 14.0.0, SAS Institute, Cary, NC, United States).

## Results

### Human and Canine Holter Recordings

After review of recordings in THEW, Holter recordings from 40 humans were studied that met the criteria. It is noted that none of the reviewed recordings in the bank had pauses > 2.5 s. Equal numbers of males and females were included with a median age of 41.7 years (range 18–80 years). Holter recordings from 130 dogs were studied. Canine recordings included 69 boxers (41 females/28 males) and 61 (32 females/29 males) non-boxers (no difference in sex distribution; *p*-value = 0.32). Of the 61 non-boxers, 31 different breeds were represented. Those with more than one dog per breed included eight mixed breed, six Shi Tzu, four Labrador retrievers, four miniature schnauzers, three great Danes, three Doberman pinschers, and two dachshunds. Boxers were overrepresented because of the high number of screenings performed for breeding animals; therefore, boxers and non-boxers were compared. The age and weight data are shown in Table 1A. The boxers were significantly younger and larger than the non-boxers.

**TABLE 1:** Heart rate and heart rate variability comparisons.

**(A)** Comparisons of populations and 24-h heart rate characteristics boxer versus non-boxer (31 other dog breeds represented). Data shown as median (range) and *p*-value from Wilcoxon Test with Bonferroni correction.			

**Parameter**	**Boxer (*n* = 69)**	**Non-boxer (*n* = 61)**	***p*-value**

Age	5(1−12)	9(1.5−16)	<0.0001
Weight (kg)	27(16−38)	15(4−61)	<0.0001
Heart rate (bpm)	81(66−112)	82(64−119)	1.0
Minimum heart rate (bpm)	40(29−59)	41(32−69)	1.0
Maximum heart rate (bpm)	240(182−333)	236(163−301)	1.0
Heart rate < 50 (min)	27(0−312)	3(0−432)	1.0
Heart rate > 120 (min)	102(16−444)	90(0−726)	1.0
Longest pause (s)	3.2(2−5.5)	2.6(0−5.5)	<0.001
Number of pauses > 2 s	157(2−2737)	40(0−2082)	<0.001

**(B)** Comparisons of 24-h heart rate dog versus human heart rate. Data shown as median (range) and *p*-value from Wilcoxon Test with Bonferroni correction.			

	**Dog (*n* = 130)**	**Human (*n* = 40)**	***p-*value**

Heart rate (bpm)	81(64−119)	74.5(59−103)	0.005
Minimum heart rate (bpm)	41(29−69)	51(34−67)	<0.001
Maximum heart rate (bpm)	236(163−333)	132(98−187)	<0.001

**(C)** Comparisons of 24-h time domain heart rate variability between boxers, non-boxers, and humans. Heart rate variability parameters corrected for heart rate (see text). Data shown are mean and standard deviation. Groups with letter (a–c) in common are not different from each other at *p* > 0.05.			

	**Boxer (*n* = 69)**	**Non-boxer (*n* = 61)**	**Human (*n* = 40)**

SDNN (ms)	0.457^(0.056)a^	0.396^(0.008)b^	0.176 (0.010)^c^
SDANNIn (ms)	0.376^(0.054)a^	0.314^(0.079)b^	0.077 (0.022)^c^
SDANN (ms)	0.249^(0.046)a^	0.215^(0.068)b^	0.157 (0.048)^c^
RMSSD (ms)	0.475^(0.075)a^	0.412^(0.132)b^	0.045 (0.021)^c^

### Hour Sinus Rate

#### Heart Rate Human Versus Dog

**FIGURE 1:**
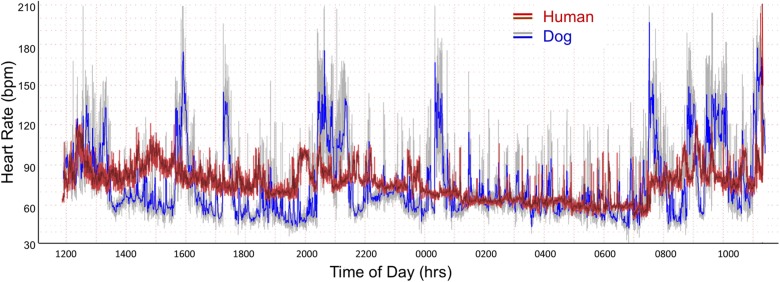
Heart rate tachogram from a human (red/magenta) and dog (gray/blue). The representative patterns exhibited in these two subjects were similar to all. The tachogram shows the heart rate over the course of a 24-h day. The inner magenta (human) and blue (dog) colors indicate the average heart rate as determined by a rolling average of 8 beats. The red (human) and gray (dog) colors indicate the maximum and minimum heart rate during that time. Between the hours of 0100 and 0730 a nocturnal dip (slowing of heart rate) is seen in both the human and dog. In the dog other periods have a similar heart rate slowing associated with sleep as indicated by the diary kept by the owners. The dog has a wider range of heart rates. From the two-dimensional heart rate tachogram the beat-to-beat distribution cannot be determined.

As shown in Table 1A the only significant differences between boxers and non-boxers concerning heart rate were the number of sinus pauses > 2 s and the duration of the longest pause with boxers having more sinus pauses and the longest pause of greater duration. The average, minimum and maximum heart rates of the dogs were compared to humans (Table 1B). All variables characterizing sinus heart rate were significantly different between dogs and humans. Although the average heart rate in the humans was slower, the spread of heart rate was greater in the dog with lower minimum and higher maximum heart rate. Figure 1 illustrates these characteristics of the sinus heart rate in the human and dog.

### Hour Heart Rate Variability

#### Heart Rate Variability Boxer, Non-boxer, and Human

Table 1C shows the difference between groups of 24-h time domain heart rate corrected indices. All groups were different from each other; however, the values in humans were markedly lower than in all dogs.

#### Heart Rate Variability and Age

Supplementary Table 1 shows the relationship of age to heart rate and each of the 24-h time domain (heart rate corrected) indices of heart rate variability. Higher heart rate was associated with advancing age in the non-boxers. However, this group of dogs had a wider age range with numerous geriatric animals when compared to the boxers. In this population of humans relationship to age was not identified. However, time domain parameters were significantly related to age in humans. No relationship of age to time domain parameters were found in the dogs.

### Histogram Beat-Interval Distribution

#### R–R Interval Histograms Human Versus Dog

**FIGURE 2:**
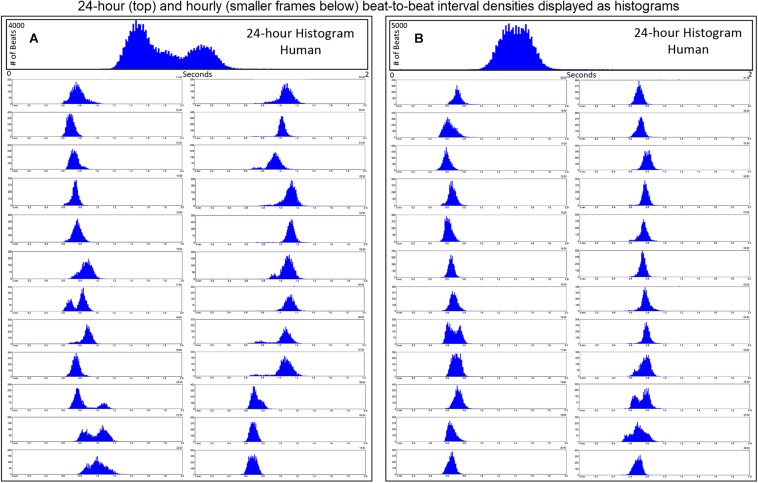
R–R interval histograms from 24-h ECG recordings from two different humans. All of the human R–R interval histograms were either similar to these two individuals or a mixture of these two types of distributions. The top expanded frame is the summation of all R–R intervals over the 24 h. Note the different density distribution of R–R intervals (thus, heart rates) in these two individuals. The individual in frame **(A)** has more time at longer R–R intervals compared to the individual in frame **(B)**. Below the 24-h summary frames the corresponding 1-h histograms for each individual are shown. The summation of these 24 single 1-h histograms form the composite shown at the top for each individual. In general, depending on physiologic needs, the heart rate over a 1-h period primarily has a Gaussian-shaped distribution. Some hours have more than one Gaussian-shaped distribution. This type of distribution exists likely because the heart rate decreases and increases linearly.

**FIGURE 3:**
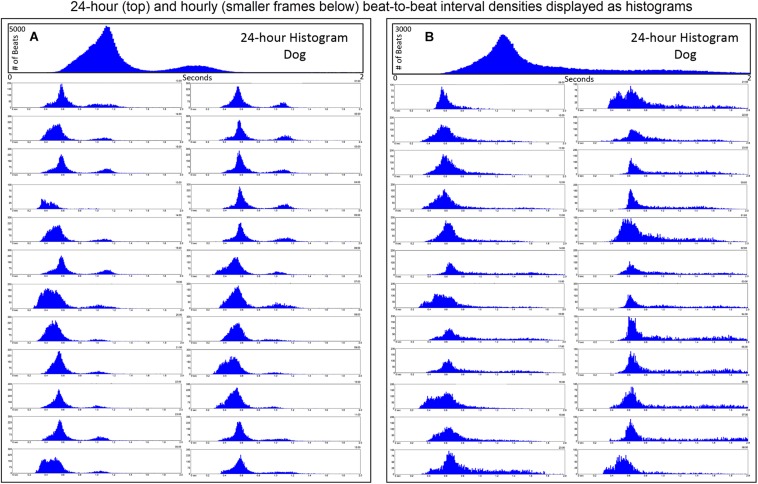
R–R interval histograms from 24-h electrocardiographic recordings from two different dogs. These two dogs represented the basic patterns seen in all dogs. The top expanded frame is the summation of all R–R intervals over the 24-h. Note the different density distribution of R–R intervals (thus, heart rates) in these two examples. Below the 24-h summary frames, the corresponding single 1-h histograms for each dog are shown. The summation of these 24 single 1-h histograms form the composite shown at the top for each dog. Note the more complicated beat distribution of the dog when compared to the human. Dog **(A)** tends to have different distributions within a given hour. Shorter R–R intervals (more leftward distribution) tend to merge with the dominant distribution centered at approximately 550 ms (109 bpm) with a paucity of beats centered at approximately 800 ms (75 bpm). A small number of beats (but not time spent at these intervals) with the distribution centered at approximately 1050 ms (57 bpm) is seen. Dog **(B)** has a dominant distribution of R–R intervals centered at 600 ms (100 bpm) with a tail of more diffusely distributed long R–R intervals. Dog **(A)** was a boxer and dog **(B)** a Doberman pincher.

Further examination of heart rate/R–R interval distribution using histogram plots of R–R intervals showed differences between humans and dogs. Figure 2 shows representative distributions in two humans that were similar to all recordings. The 24-h summation is a composite of R–R intervals shown for each hour. A shifting left or right of the interval densities approximates a Gaussian distribution. In contrast, Figure 3 shows examples of two dogs with typical histogram distributions of R–R intervals. The 24-h summed distributions are not from shifts of singular shifting Gaussian patterns, but instead are from bimodal or trimodal distributions and skewed R–R intervals to the right/longer intervals. These patterns were seen across all breeds of dogs. Singular Gaussian patterns were seen in dogs during hours with documented activity or the hour during application or removal of the Holter recorder. The more common bimodal distributions corresponded to time-periods with the most distinct zone of avoidance and during the sleep hours.

#### Singular Versus Bimodal Histographic Distribution in the Dog

Time domain and frequency domain heart rate variability were used to determine if the bimodal versus the singular interval distribution was associated with greater parasympathetic modulation as qualitatively judged by heart rate variability for each of 128 dogs. To accomplish this, hours were selected with the greatest (bimodal) and least (singular) paucity of R–R intervals. Singular distributions were during the Holter application. Hours with the bimodal distribution had greater heart rate variability as judged by time domain parameters and greater power density overall. The latter was the result of markedly higher high frequency power (Table 2A). During a given hour or 24-h, an additional beat distribution at short R–R intervals associated with activity could be identified making a trimodal distribution. Histographic representation of these shorter R–R intervals often overlapped with the largest density that was characterized by a distribution that centered around 600 ms (Figure 3). The longer R–R interval distribution was either a Gaussian-shape distribution or flat with greater range of intervals. These two types of distributions were further evaluated (see below).

**TABLE 2:** Comparisons of bimodal versus singular histogram distributions within dog and clustered versus branched beat-to-beat interval patterns between dogs.

**(A)** Within dog (*n* = 128) (paired data) comparison of an hour with a large paucity of beats resulting in a bimodal distribution and an hour with a small paucity of beats characterized as a single distribution. Heart rate variability parameters corrected for heart rate (see text). Data shown as median (range) and *p*-value from Wilcoxon Signed Rank Test with Bonferroni correction.			

**Parameter**	**Bimodal**	**Singular**	***p*-value**

Average heart rate (bpm)	68(50−130)	110(75−163)	<0.001
Beat-to-beat interval (ms)	882(462−1200)	546(368−800)	<0.001
Minimum heart rate (bpm)	48(32−139)	69(34−108)	<0.001
Maximum heart rate (bpm)	147(80−236)	199(123−313)	<0.001
SDNN (ms)	0.38(0.22−0.58)	0.27(0.07−1.35)	<0.001
SDANNIn (ms)	0.34(0.15−0.49)	0.20(0.05−0.55)	<0.001
SDANN (ms)	0.11(0.03−0.50)	0.16(0.03−0.59)	0.032
RMSSD (ms)	0.47(0.18−0.69)	0.24(0.04−0.57)	<0.001
Frequency domain total power (ms^2^)	24486(1379−66913)	4493(335−31063)	<0.001
Frequency domain HRV-HF (ms^2^)	19311(33−59924)	312(14−10770)	<0.001

**(B)** Comparison of beat patterning (clustered versus branched) during a single sleep hour in 50 dogs (13/12 boxers/non-boxers each group). Heart rate variability parameters corrected for rate/interval (see text). Data shown as median (range) and *p*-value from Wilcoxon Test with Bonferroni correction.			

	**Clustered (*n* = 25)**	**Branched (*n* = 25)**	***p*-value**

Average heart rate (bpm)	71(57−93)	54(46−92)	<0.001
Beat-to-beat interval (ms)	845(645−1053)	1111(652−1304)	<0.001
Minimum heart rate (bpm)	55(42−68)	41(34−71)	<0.001
Maximum heart rate (bpm)	144(103−196)	137(112−174)	0.99
Number of pauses	1(0−16)	24(0−374)	0.006
SDNN (ms)	0.349(0.156−0.468)	0.409(0.273−0.467)	0.008
SDANNIn (ms)	0.312(0.128−0.424)	0.369(0.245−0.426)	0.02
SDANN (ms)	0.099(0.049−0.205)	0.118(0.038−0.226)	0.36
RMSSD (ms)	0.477(0.167−0.619)	0.481(0.373−0.686)	0.99
Frequency domain total power (ms^2^)	19036(6352−59061)	33541(11492−86423)	<0.001
Frequency domain HRV-HF (ms^2^)	15856(4107−55894)	19741(5205−44501)	0.2

#### R–R Interval Tachograms Human Versus Dog

**FIGURE 4:**
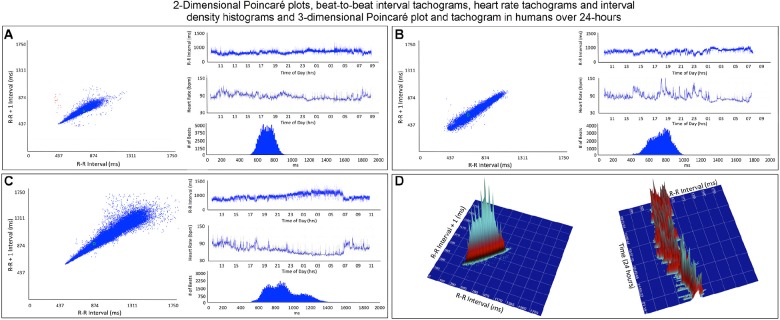
This figure illustrates in four humans the beat-to-beat relationship shown by two-dimensional Poincaré plots and the beat distribution identified by R–R interval tachograms, heart rate tachograms and R–R interval histograms. The recording in frame **(A)** shows an individual with the fastest average heart rate (85 bpm) (red dots indicate low numbers of ventricular premature complexes) and the recording in frame **(C)** shows an individual with the slowest average heart rate (67 bpm). These three recordings **(A–C)** illustrate that although the density of R–R intervals (heart beat distribution) varies from fast to slow depending on physiologic needs, the heart rate in humans tends to change linearly along the line of identity. In some, the spread of intervals increases at longer intervals. Two-dimensional Poincaré plots and R–R interval tachograms do not provide an appreciation of the number of beats at a given interval. In frame **(D)**, three-dimensional plots of the R–R intervals from a 24-h recording from a normal human are shown as a histographic Poincaré plot (left) and tachogram (right). These type of plots provide an appreciation of the beat density for a given R–R interval on a beat-to-beat basis (histographic Poincaré plot) or over the course of time (three-dimensional interval tachogram). The amount of time examined can be selected(http://wyattflanders.com/poincare/ and http://wyattflanders.com/poincareplot/).

**FIGURE 5:**
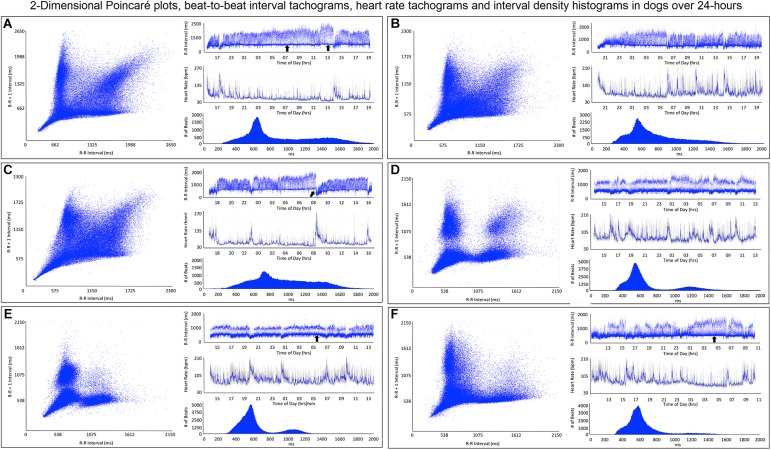
This figure illustrates in six dogs the beat-to-beat relationship shown by two-dimensional Poincaré plots and beat distribution identified by R–R interval tachograms, heart rate tachograms and R–R interval histograms. All dogs had these types of the distributions. It is important to realize that on a two-dimensional plot each of the R–R intervals, when they are the same, they overlay each other such that the beat density (actual number of beats at a particular interval) are only partially appreciated. The geometric images of heart rate variability illustrate the difference in the distribution of heart beats not only between dogs and humans (compare to Figure 4), but also amongst dogs. When the dog is compared to the human, the beat distribution characteristics include: (1) Slowing of the heart rate that is non-linear after a bifurcation, such that heart rate changes are not restricted to the line of identity (diagonal line from lower left corner to upper right corner). (2) Wider distribution of R–R intervals with a varying degree of a paucity of beats (‘zone of avoidance’) identified in the Poincaré plots, R–R interval tachograms and R–R interval histograms. The ‘zone of avoidance’ cannot be appreciated from the heart rate tachogram. The similarities among the dogs in the Poincaré plots include a ‘stalk’ showing faster beat-to-beat intervals likely because of higher sympathetic modulation and lower parasympathetic modulation. The beat-to-beat distribution spreads/bifurcates when the R–R interval extends beyond approximately 600 ms. As illustrated in frames (**A–F)**, dogs differed in the Poincaré plots by the degree of beat-to-beat ‘branching’ versus the amount of beat-to-beat ‘clustering,’ the paucity of beat-to-beat intervals and the appearance of long–long intervals resulting in a ‘cloud.’ When the branches are parallel to the axes this indicates that as the long interval increases the short interval stays more constant (frame **A**). When the arm deviates up on the x-axis or right on the Y axis this indicates that the short intervals are increasing as the long intervals increase (frame **C**). Frames (**A–C)** show faster beat-to-beat relationships that with slowing results in branching that spreads as wide bands with the long–short/short–long relationship of sinus arrhythmia. Additionally, a region of long–long R–R intervals shown as a ‘cloud’ extends with an upper border along the line of identity. Of these three dogs (**A–C**) the one in frame **(A)** has the most obvious region with a paucity of R–R intervals. Frames (**D–F)** show a more distinct zone of avoidance. Additionally, **(D,E)** have beat clustering evident not only in the Poincaré plots but also throughout the R–R interval tachograms. Also evident on the R–R interval tachogram is the appearance of a thick line (broad black arrows in frames **A,E,F**). The R–R intervals appear to protrude downward with sharp spikes from this relatively constant ‘line’ that at approximately 600 ms. The R–R interval of this line approximated the bifurcation interval (see Table 3) seen on the Poincaré plots. Note in frame **(C)** that the dog suddenly has an increase in heart rate (decrease in R–R interval) indicated with the broad angled black arrow. This was due to sudden excitement noted on the diary. For the rest of the Holter the paucity of beats (zone of avoidance) is less apparent during a period of changing heart rate. See text and additional figures for explanation.

R–R interval tachograms showed consistent differences between humans and dogs (Figures 4, 5). The patterns shown in these figures represent those that were seen for all subjects. Each dot represents an R–R interval. Tachograms in humans had the appearance of a narrow band that would move up and down as the intervals lengthened and shortened (Figure 4). In contrast, the patterns in dogs were more complex. Three general features characterized the tachograms of the dogs (Figure 5):

(1) Broader spread of longer intervals. This region of longer intervals varied in its spread between dogs or within dog depending on the wake-sleep cycle. The greatest spread occurred during documented sleep.
(2) Regions of lower beat density (infrequent R–R intervals) that looked like a ‘white-band’ separating shorter and longer R–R intervals. Often this low beat density band was consistent over the day (this region identified as the zone of avoidance). Two general characteristics were noted: (1) low beat density separated by two distinct wide bands of R–R intervals and (2) region of lower beat density with more diffuse band at longer R–R intervals. During times of heart rate change, these banding characteristics were less distinct.
(3) A denser region of shorter R–R intervals that appeared as a ‘dark-band’ that also was consistent over the day. This band tended to have the appearance of a ‘line’ demarcating the shortest intervals. Shorter intervals did interrupt this ‘line’ and correlated with abrupt increases in heart rate (shorter intervals), and often associated with artifact indicating body movement (exercise/excitement).

### Beat-to-Beat Analyses: Poincaré Plots

#### Two-Dimensional Poincaré Plots

To gain a better understanding of the unique patterns identified in the dog when compared to the human, beat-to-beat analyses were performed. As shown in Figures 4, 5, the two-dimensional Poincaré plots of the human and dog are distinctly different. In the human, as the heart rate changes with shorter to longer intervals the beat-to-beat changes occur along the line of identity of the Poincaré plot. In Figure 4D a three-dimensional histographic Poincaré plot more clearly illustrates the beat-to-beat distribution along the line of identity and the three-dimensional tachogram shows the change in beat intervals throughout the day. Importantly, is the notation that these graphs show number intervals; however, this does not give a representation of the amount of time and at shorter versus longer intervals. That is, the lower density of the longer intervals should not be interpreted as minimal or infrequent because the ‘time spent’ may be greater. In contrast, two-dimensional Poincaré plots in the dog (Figure 5) illustrate that beat intervals occur along the line of identity until a point at which a bifurcation occurs resulting in a wide spread corresponding to long–short and short–long intervals. In the dog, two-dimensional Poincaré plots could be described as branched or clustered (see section “Materials and Methods”) with a paucity of interval beats of varying degree. Because these plots were a summation of the entire 24-h, it was necessary to examine shorter times to understand the formation of these patterns.

#### Dynamic Poincaré Plots

**FIGURE 6:**
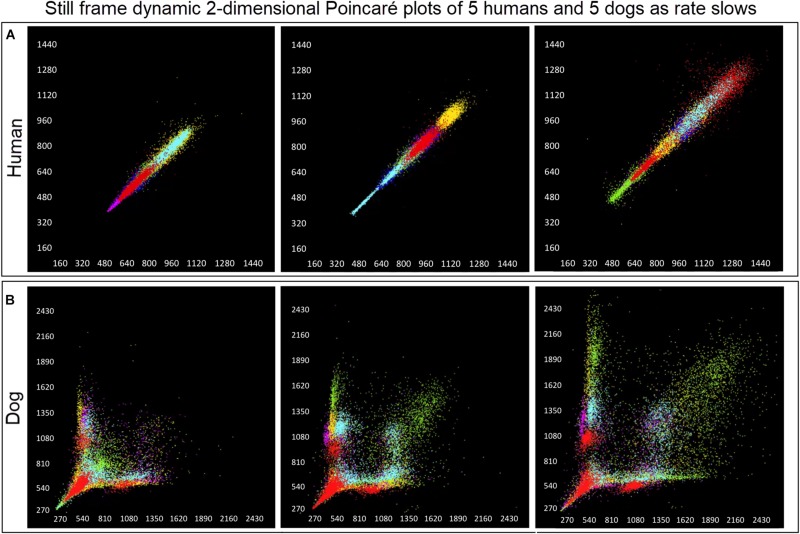
(Supplementary Videos 1, 2) Examination that is limited to still frames of two-dimensional geometric heart rate variability restricts understanding of the beat-to-beat dynamics. Here, frames captured from dynamic/animated two-dimensional Poincaré plots of five humans **(A)** and five dogs **(B)** provide the ability to examine heart rate dynamics in the human and dog.http://wyattflanders.com/poincare/ Each individual dog or human is represented by a different color. The R–R interval (X axis) and R–R + 1 interval (Y-axis) (intervals are in ‘ms’) are plotted with the ability to change the number of intervals represented (for clarity, labeling for these axes is omitted). The associated videos (Supplementary Videos 1, 2) for these frames illustrate the difference between the dog and human concerning the beat-to-beat increasing and decreasing of heart rate and associated beat-to-beat changes. In the human, these changes occur primarily along the line of identity (diagonal line from lower left corner to upper right corner). In the dog as the beat-to-beat intervals increase they do so along the line of identity until approximately 600 ms when a bifurcation develops resulting in two ‘branches’ or ‘clusters’ along the X and Y axes. In the dog, a paucity of beats is noted for certain beat-to-beat intervals (zone of avoidance). The latter will not be evident if an excessive number of beats are shown. Additionally, some dogs develop a ‘cloud’ of long–long R–R intervals with an upper border along the line of identity. These videos can also have lines added and the speed adjusted so that the exact beat-to-beat interval relationships are seen (see Figure 8 and Supplementary Videos 3–6). Supplementary Videos 1, 2 correspond with this figure.

**FIGURE 7:**
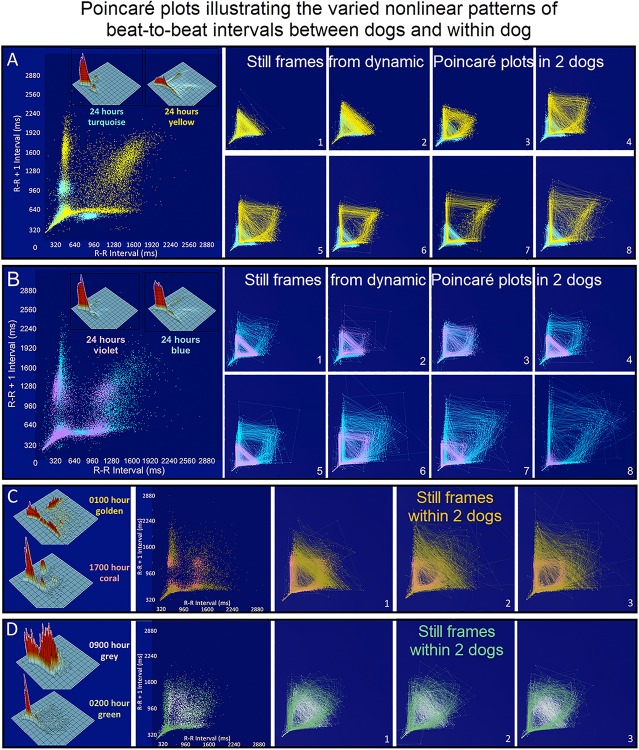
(Supplementary Videos 3–6) Still frames from dynamic Poincaré plots (http://wyattflanders.com/poincareplot/) of six different dogs demonstrate the varied R–R interval patterns between dogs (frames **A,B**) and within dogs (frames **C,D**). In frames **(A,B)** large Poincaré plots without connecting lines contain 10,000 beat intervals and above this plot, the corresponding three-dimensional histographic Poincaré plots of the entire 24-h are shown. Below the three-dimensional histographic plots, a color code is stated for that dog that corresponds with the animated Poincaré plot in this figure and in the accompanying Supplementary Videos. To the right of this panel, from the dynamic two-dimensional Poincaré plots still frames of 1000 intervals with lines, the beat-to-beat relationships are presented. The still frames were captured at different time points during 24-h electrocardiographic recordings (Frames **1–8**). In frame **(A)**, the dog in aqua (boxer) has a clustered pattern with no long–long intervals while the dog in yellow (small mixed-breed) has a long-branched pattern with a long–long R–R interval cloud. Compare the beat density of the dog in yellow (Frame **A**) to the dog in blue (Frame **B**). In frame **(B)** a boxer with a clustered pattern (color violet) also has the central cloud of long–long intervals. The Doberman pinscher in blue shows a large variation in long–long intervals. Frames **(C,D)** illustrate the varied patterns within dog. The hour of the day and color representing that hour for the dynamic Poincaré plots are indicated to the right of the three-dimensional histographic Poincaré plot for each dog. The pattern of sinus arrhythmia can change over time with varying autonomic input. This also shows that if the entire 24 h is considered at one time the true dynamics of the interval relationships can be masked. The dog in frame **(C)** (boxer) during the sleep hour 0100 (golden) has a ‘branched’ pattern with slower rates; however, a more clustered pattern with faster rate is identified during an hour of wakefulness (coral). The dog in frame **(C)** (Wheaton Terrier) during the 0900 h (gray) was noted to have wide swings in heart rate associated with different activities as documented in the diary. This resulted in many transitions of rate with a more dense pattern; however, during the sleep hour (green) a different pattern was identified with the zone of avoidance evident. Supplementary Videos 3–6 provided with this figure.

**FIGURE 8:**
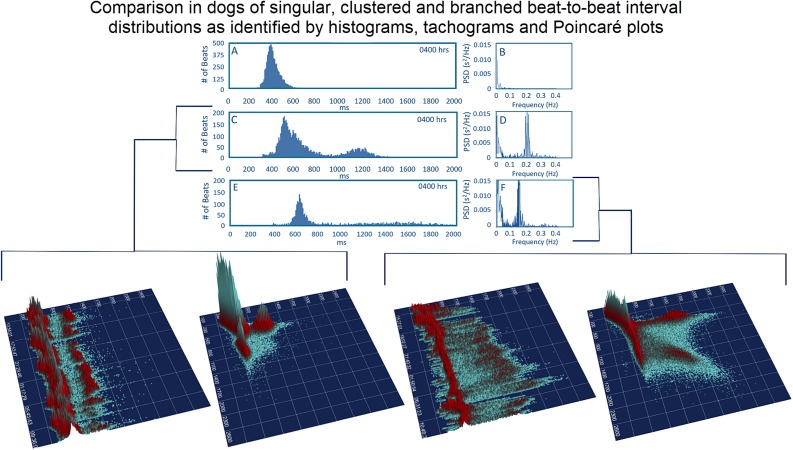
To assist in understanding some of the geometric patterns identified in the dog analyses were performed after visual inspection to categorize major beat-to-beat patterns. Initially, the hourly R–R interval histograms were inspected to identify those with a single Gaussian-shaped distributions and those with two or more distributions apparent that also had a region with a paucity of beats (Frames **A,C,E**). Statistics regarding time domain and frequency domain characteristics are provided in Table 2. In this figure, examples of the two-dimensional histogram and corresponding power spectrum/frequency domain profile of heart rate variability (Frames **B,D,F**) are shown. The three-dimensional R–R interval tachograms and histographic Poincaré plots for the two different dogs shown in frames **(C,D)** and frames **(E,F)** are bracketed below. Each of these dogs was a boxer.

**FIGURE 9:**
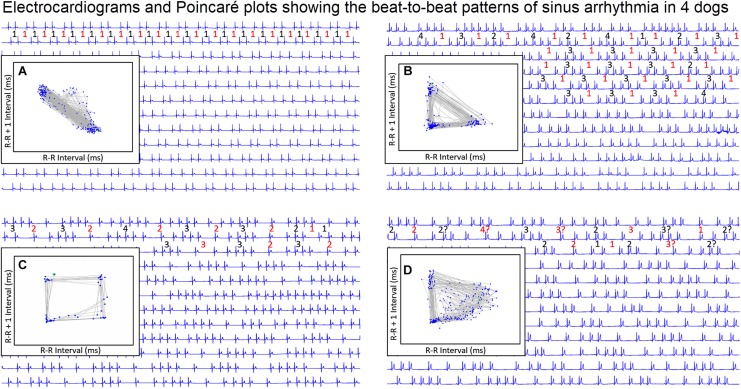
During stable sleep hours, focused intervals were identified on the electrocardiogram and compared to the Poincaré plots. This figure illustrates four common patterns. Each line is 30 s. The inset is the Poincaré plot of the ECG frame shown (for clarity the specific R–R interval in milliseconds is omitted). Frame **(A)** shows the Poincaré plot when the sinus arrhythmia has primarily a ratio of one-short R–R interval to one-long R–R interval. Because there are no consecutive short-short R–R intervals, no clusters of intervals are seen in the lower left region of the Poincaré plot. Frame **(B)** shows the Poincaré plot when the primary ratio is one-long R–R interval to two to four-short R–R intervals. The short-short intervals are in the lower left region and the single long interval between the clusters of short intervals results in a triangular shape (long–short interval followed by short intervals followed by short–long interval). Frame **(C)** shows the pattern when the sinus arrhythmia has primarily two-long R–R intervals with clusters of short intervals. The long–long intervals result in a cloud in the upper right region beginning at the line of identity. The resulting shape is that of a polygon. Frame **(D)** illustrates the shape of the Poincaré plot with more variability in the R–R intervals creating a long–long cloud of intervals. From frame **(D)** the R–R intervals before the speeding of the rate are more variable than the long R–R interval that follows the short sequence. That is, with sinus arrhythmia the long interval after the faster rate is more consistent then the long interval before the faster rate. It is important to note that these examples were taken during a time when each of the dogs was displaying a sinus arrhythmia with presumed high parasympathetic tone and not interrupted by marked increases in sympathetic tone. Without a sympathetic surge note that none of the short-short intervals goes below a specific point. Although for clarity the scale is omitted, this was approximately 600 ms. This corresponds to the limit of the R–R interval seen on the R–R interval tachogram (see Figure 7 and Table 3). For each frame, the numbers in red are long R–R intervals and numbers in black are short R–R intervals. If a ‘?’ follows the number, this indicates that some uncertainty exists concerning the exact number of intervals defined as long or short (e.g., R–R interval duration is medium).

Dynamic (animated) Poincaré plots were developed to examine both human and canine data sets (Figure 6 and Supplementary Videos 1, 2). This new methodology illustrated the difference in the increasing and decreasing of heart rate on a beat-to-beat basis between the human and dog. Humans consistently changed rate in a linear fashion, but dogs had a visual bifurcation at a relatively stable point on the graph. However, the spread of beat intervals after the bifurcation point differed amongst the dogs (Figures 7, 8). Examination of individual hours with lines connecting each successive beat interval provided the ability to see the sequence of heartbeat intervals (Figure 9). From these data it was apparent that dogs had different patterns after the bifurcation from the consistent shorter intervals (Figures 7A,B and Supplementary Videos 3, 4). Moreover, an individual dog could have different patterns throughout the day (Figure 7C and Supplementary Videos 5, 6). The dynamic Poincaré plots with lines illustrating sequence clearly showed the paucity of beats/zone of avoidance for both clustered and branched R–R interval distributions.

#### Three-Dimensional Tachograms and Histographic Poincaré Plots

Three-dimensional plots revealed a truer representation of beat distributions (Figures 4, 8) permitting selection of 25 clustered and 25 branched R–R interval patterns (Figure 8). Time domain and frequency domain analyses of heart rate variability revealed slower heart rates and mixed evidence of greater parasympathetic modulation with beat distribution characterized as branched versus clustered (Figure 8 and Table 2B). Beats clustered more with a sequence of short–long intervals. That is, the last beat sequence of short–long intervals was more consistent than that of a long–short interval. When examining these plots it is important to keep in mind that the interval density mathematically will show a greater density for the short intervals rather than the longer intervals, but this does not reflect the ‘time’ the heart spent at shorter or longer rates.

### Electrocardiographic Relationship to Beat-to-Beat Patterns

#### Beat-to-Beat Pattern With Slowing Heart Rate

**FIGURE 10:**
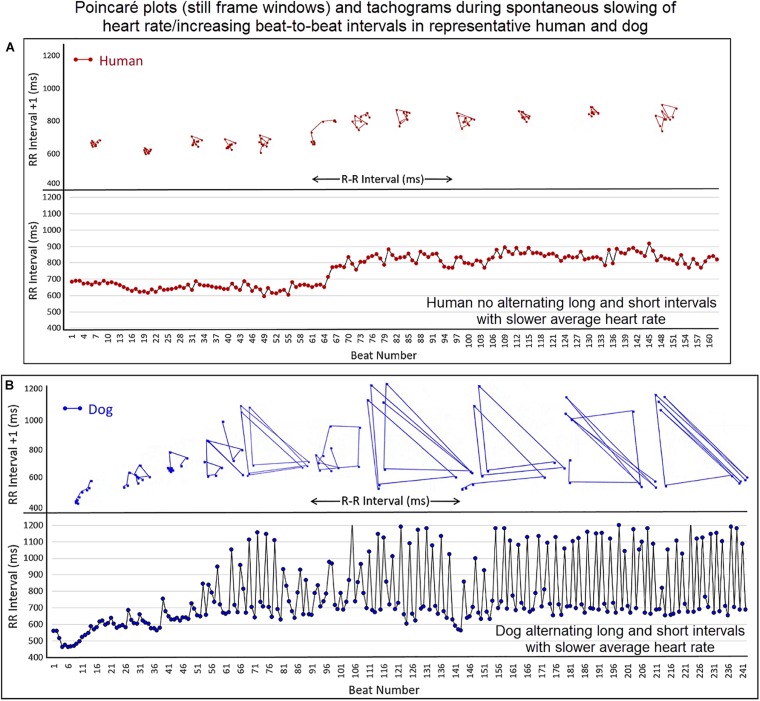
(Supplementary Video 7) This figure illustrates the slowing of the sinus rhythm in the human and dog. As the human sinus node slows the beat-to-beat variation is confined along the line of identity (Frame **A**). As the dog sinus node slows (Frame **B**), the rate slows along the line of identity, just as in the human, until approximately 600 ms when a bifurcation occurs with long–short R–R intervals. This is shown in the Poincaré plots as well as the tachogram in this focused illustration. For clarity, the scale for Poincaré plots on the x-axis is not shown. Supplementary Video 7 complements this figure.

To understand the beat patterns identified in the dog the dynamic Poincaré plots were examined in conjunction with visualization of the electrocardiogram. Figure 9 illustrates this examination of four dogs during a stable sleep hour revealing the differing patterns of sinus arrhythmia and corresponding Poincaré plots. Note that during these stable sleep times with likely low sympathetic input, no short-short R–R intervals along the line of identity are present (compare with Figures 7, 8). However, the bifurcation point is consistent and is identified in the dog when the heart rate slows after a sympathetic stimulation associated with excitement (Figure 10 and Supplementary Video 7). The human heart rate slows linearly throughout the full range of beat intervals; however, although the dog heart slows initially along the line of identity when the heart rate slows to a particular interval, a bifurcation develops. This interval was coined the ‘bifurcation interval.’ An additional finding from the comparison of the Poincaré plots with the electrocardiogram is the ‘cloud’ of longer intervals. The cloud associated with long–long (Figure 5D) intervals in many dogs formed a mass effect of intervals widely spread around the line of identity.

#### Bifurcation Interval/Zone

It was noted that amongst dogs the bifurcation interval was visually within a narrow range of beat-to-beat intervals. Moreover, this bifurcation interval or zone corresponded to the same visual point identified on the tachogram as a ‘line’ of ‘usually’ shortest-intervals during stable sleep (see Figure 5). Therefore, measurement from the canine data set during a stable sleep hour in 25 boxers and 25 non-boxers was performed to determine the range for the bifurcation interval, its relationship to overall heart rate and its relationship to a measure of parasympathetic modulation using the overall time domain variable, SDNN corrected for heart rate. Data were normally distributed with results in Table 3. The average heart rate during the sleep hour was correlated modestly with the bifurcation interval and rate corrected SDNN; that is, the longer the bifurcation interval, the slower the heart rate and the higher the SDNN. The bifurcation interval had a 95% confidence interval (Table 3) that was equivalent to a heart rate range of 97.8–102.8 bpm. The bifurcation interval was not correlated with SDNN.

**TABLE 3:** Pairwise correlation of bifurcation interval measured during stable sleep hour in 50 dogs^∗^ with heart rate, R–R-interval, rate corrected SDNN, and age.

Parameter	Mean	*SD*	95% CI	
Bifurcation interval (ms)	598.5	54.3	583.5–613.5	
Age (years)	6.5	3.32	5.6–7.4	
Average heart rate (bpm)	63.6	11.1	60.4–66.7	
Average R–R-interval (ms)	971	164	924–1018	
SDNN (ms)	0.364	0.07	0.343–0.386	

**Variable**	**By variable**	**Correlation *r***	**95% CI of *r***	***p*-value**

Bifurcation interval (ms)	Age (years)	−0.102	−0.367 to 0.182	0.48
Bifurcation interval (ms)	Average heart rate (bpm)	−0.572	−0.733 to −0.349	<0.001
Bifurcation interval	SDNN (ms)	−0.235	−0.482 to 0.05	0.10
Average heart rate (bpm)	SDNN (ms)	−0.342	−0.566 to −0.070	0.02

*CI, confidence interval. ^∗^Boxers and non-boxers were combined because data for each of these variables were not different for these data.*

### Parasympatholytic and Parasympathomimetic Drug Effects

**FIGURE 11:**
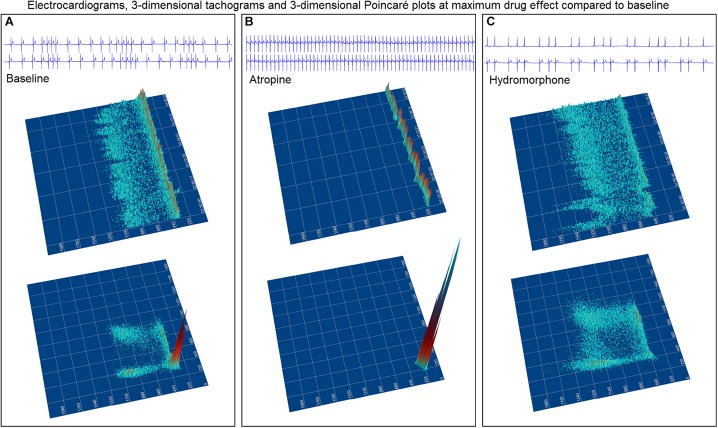
The peak effects of atropine (0.04 mg/kg given intravenously) (Frame **A**) and hydromorphone (0.2 mg/kg given intravenously) (Frame **B**) compared to baseline (Frame **C**) are shown in the electrocardiograms, three-dimensional tachograms and Poincaré plots of beagle 5. During the baseline recording the sinus arrhythmia has a pattern with a paucity of beat intervals that can be identified on both the tachogram and Poincaré plots. At the longest intervals a cloud appears near the line of identity. After the administration of atropine (Frame **B**) that results in a decrease in parasympathetic modulation, the heart rate not only increases with a loss of sinus arrhythmia but the pattern of beat-to-beat intervals becomes linear. After the administration of hydromorphone (Frame **C**) that results in an increase in parasympathetic modulation, the heart rate slows and the bifurcation interval increases with a paucity of beat intervals (zone of avoidance). Scaling of all images is the same to show proportionality.

**FIGURE 12:**
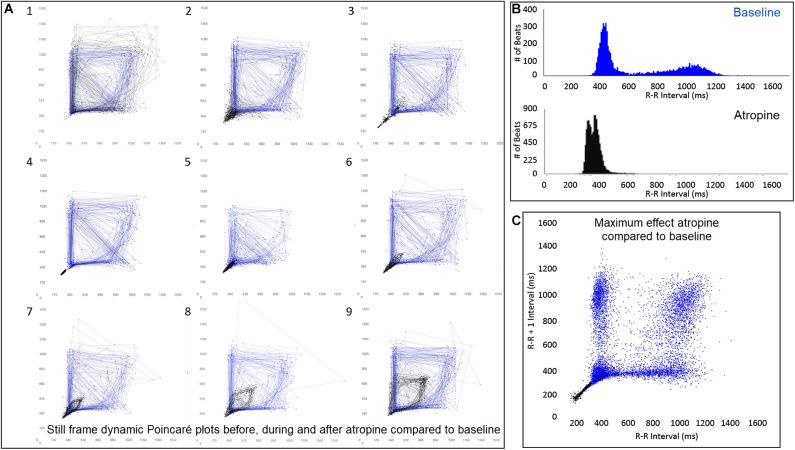
(Supplementary Video 8) Still frames from dynamic Poincaré plots (Frame **A1–9**) from the electrocardiographic recordings from two different days during a stable rest period (blue) and just before, during and after administration of atropine (black) (0.04 mg/kg given intravenously) in beagle 8. Dots represent the beat-to-beat interval (R–R interval surrogate for P–P interval with all these verified to be sinus in origin) with lines connecting the next beat-to-beat interval. Each frame shows 500 beat-to-beat intervals. After atropine, the parasympatholytic effects result in the beat-to-beat intervals becoming shorter (Frames **1–4**) and as parasympathetic modulation returns, the beat-to-beat intervals become longer (Frames **5–9**). Note that after treatment with atropine the beat-to-beat patterning characterized by a bifurcation collapses to short intervals that hug the line of identity. When the heart rate slows (longer beat-to-beat intervals) with the decreasing drug effect, the bifurcation is identified again. In frame **(B)**, the number of beats at a particular beat interval are shown for the stable rest time (blue) and during treatment with atropine (black). The double peak during the atropine treatment represents the faster rate during the intravenous injection followed by the true drug effect and its short beat-to-beat intervals. In frame **(C)**, the Poincaré plot illustrates a comparison without the connecting lines of the entire baseline period and the time of maximum effect of atropine. Supplementary Video 8 complements this figure.

**FIGURE 13:**
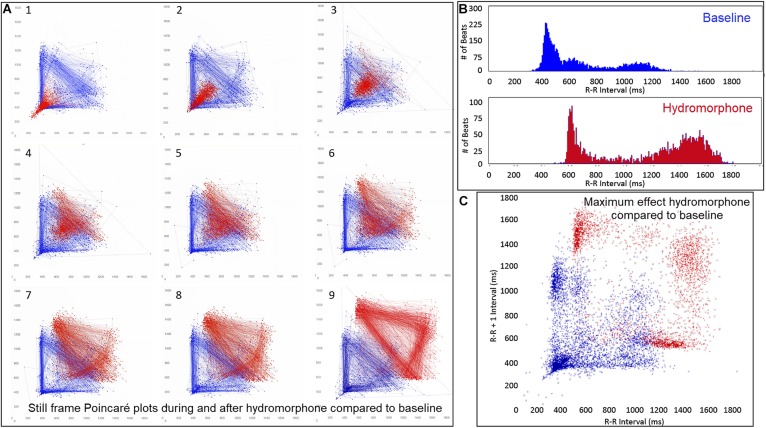
(Supplementary Video 9) Still frames from dynamic Poincaré plots (Frame **A1–9**) derived from intervals of 24-h electrocardiographic recordings from two different days during a stable rest period (blue) and just before and after administration of hydromorphone (red) (0.2 mg/kg given intravenously) given to beagle 1. Dots represent the beat-to-beat interval (R–R interval surrogate for P–P interval with all these verified to be sinus in origin) with lines connecting the next beat-to-beat interval. Each frame shows 1500 beat-to-beat intervals. After hydromorphone the beat-to-beat intervals become longer. In frames (**1–5)** the heart rate is faster because the dog is excited during the intravenous administration and then calms as the drug takes effect. The heart rate and beat-to-beat interval pattern changes with increasing drug effect. The characteristic pattern with a paucity of beat intervals (zone of avoidance) and beat intervals that do not hover around the line of identity are seen in the dog with high parasympathetic modulation induced by the hydromorphone. In frame **(B)** the number of beats at a particular beat interval are shown for the stable rest period (blue) and during treatment with hydromorphone (red). In frame **(C)**, the Poincaré plot illustrates a comparison without the connecting lines of the entire baseline period and the time of maximum effect of hydromorphone. Supplementary Video 9 complements this figure.

**FIGURE 14:**
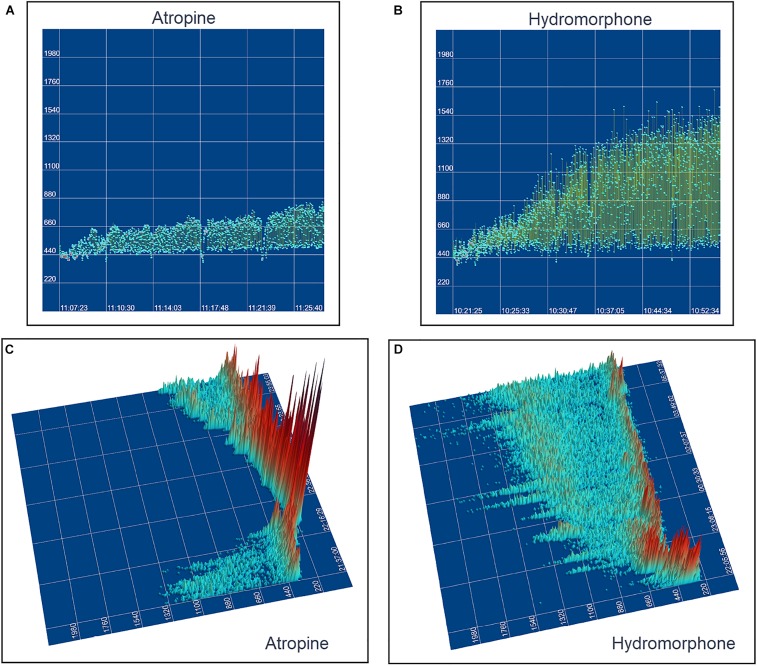
This figure uses two-dimensional **(A,B)** and three-dimensional **(C,D)** tachograms to show the pattern and density of sinus intervals before, during, and after treatment to decrease and increase parasympathetic modulation. Recordings were from two different days for each treatment. Frame **(A)** (beagle 1) illustrates the interval changes (turquoise dots represent interval duration and yellow lines connect sequential intervals) as the drug effects of atropine (0.04 mg/kg given intravenously) dissipate. The decreasing and increasing interval duration associated with the increasing and decreasing drug effects of atropine are seen in frame **(C)** (beagle 1). As the effects of atropine subside the bifurcation interval increases with an increased paucity of beats as sinus arrhythmia returns. Frame **(B,D)** (beagle 8) show increasing interval duration associated with the increasing drug effects of hydromorphone (0.2 mg/kg given intravenously). The effects of hydromorphone result in an increased paucity of beats, an increase in the bifurcation interval and a greater spread of long intervals. Scaling for frames **(C,D)** are equal to show proportionality of the interval density.

The beat-to-beat patterns after both atropine and hydromorphone were compared to baseline during and following the peak of drug effects (Figures 11–14 and Supplementary Videos 8, 9). After treatment with the parasympatholytic drug atropine, the heart rate increased (decrease in beat-to-beat interval) as expected. Additionally, the beat-to-beat interval did change below the identified bifurcation interval in a linear fashion. Over time, dynamic Poincaré plots and tachograms revealed that as the parasympatholytic effects waned a bifurcation and non-linear slowing of the heart rate (increase in beat-to-beat interval) developed (Figures 11, 12, 14 and Supplementary Video 8). Treatment with hydromorphone with its parasympathomimetic effects (Deo et al., 2008) not only showed a slowing of rate with an increase in the beat-to-beat interval, but also a loss of linear heart rate changes. The zone of avoidance or paucity of beats as seen on the Poincaré plots was expanded (Figures 11, 13, 14 and Supplementary Video 9). Each of the features described above was noted in all eight dogs.

## Discussion

The present study investigated the unique beat-to-beat patterning of sinus rhythms in the dog compared to the human. To accomplish the study objectives, additional tools of geometric beat-to-beat analyses were developed. The major findings in this study include: (1) During sinus arrhythmia the dog has a unique non-Gaussian and non-linear patterning when compared to humans as revealed by interval distributions (histograms and tachograms) and beat-to-beat maps (two- and three-dimensional and dynamic Poincaré plots). (2) Dogs have distinctive beat-to-beat distributions with regions of low beat density (zone of avoidance) and patterns (clustered and branched) associated with potentially different parasympathetic and sympathetic influence as reflected by qualitative assessment of time and frequency domain indices of heart rate variability. Furthermore, administration of parasympatholytic and parasympathomimetic drugs supported the role of the parasympathetic system in dictating the patterns identified. (3) The patterns of beat-to-beat variability in the dog evidenced by the dynamic Poincaré plots revealed a consistent region or zone (bifurcation interval) at which the long- and short- intervals of sinus arrhythmia became non-linear. Moreover, the results of this study in a clinical population of dogs under the influence of spontaneous changes in autonomic input, is congruent with the hypotheses of experimental canine studies (Fedorov et al., 2009, 2010; Glukhov et al., 2013; Lou et al., 2013, 2014; Kalyanasundaram et al., 2019) of the sinus node that demonstrate the likelihood of parasympathetic influence on the sinoatrial conduction pathways (SACPs). Finally, these studies serve as a background to the potential understanding of not only normal sinus node function in the dog, but of potential mechanisms of sinus node dysfunction.

### Different Ways to Speed and Slow

Each of the geometric heart rate indices used in the assessment of beat-to-beat changes in rate showed clear differences between the human and dog. The unique pattern identified in the dog may be related to key structural components of the sinus node complex and the electrophysiologic consequences of parasympathetic modulation and how these target the key receptors of spontaneous depolarization of pacing cells and the exit to the surrounding atrial tissue. Numerous investigations of structure and function of the sinus node conclude a similarity between the human and dog (Fedorov et al., 2009, 2010, 2012; Nikolaidou et al., 2012; Csepe et al., 2015, 2016; Kalyanasundaram et al., 2019) however, the influence of vagal modulation may be more profound in the dog. In contrast to smaller species (e.g., mouse, rabbit) with a thinner atrium and a sinus node that functions more similarly to a two-dimensional structure, the canine and human have a three-dimensional structure (Fedorov et al., 2009, 2010, 2012; Nikolaidou et al., 2012; Li et al., 2017). In the larger hearts, specific SACP connect the sinus node to the atrium (Fedorov et al., 2009, 2010, 2012; Nikolaidou et al., 2012; Glukhov et al., 2013; Lou et al., 2014; Csepe et al., 2015; Li et al., 2017; Kalyanasundaram et al., 2019). These discrete exit pathways (2–5 in the dog) have been identified by thorough investigations of structure and function using high-resolution optical mapping, action potential morphologies, immunostaining and histologic confirmation (Opthof, 2000). Because these narrow SACP slow the impulse velocity from the pacing cells, charge accumulates to overcome the source-sink mismatch between the sinus node and atria. These studies and others have demonstrated that the stimulation of the heart beat is the result of not only the pacing cells within the complex compartmentalized sinus node, but also dependent on the conduction of these impulses reaching the atrial myocardium through the SACP. Just as the spontaneous depolarization rate shifts in the location of the leading sinus pacing cells, SACP are substantially influenced by mediators of autonomic tone. For example, depending on the dose of adenosine or acetylcholine, not only are the membrane (I_f_ current) and voltage (Ca^2+^) clocks suppressed to slow depolarization (Gao et al., 2010; Lou et al., 2013; Li et al., 2017), but conduction through the SACP is slowed (Opthof, 2000; Fedorov et al., 2009, 2010, 2012; Nikolaidou et al., 2012; Glukhov et al., 2013; Lou et al., 2013, 2014; Kalyanasundaram et al., 2019). Exit block through the SACP can develop in the dog with high levels of acetylcholine corresponding to high vagal tone potentially obtained during sleep (Glukhov et al., 2013; Kalyanasundaram et al., 2019). Although the intrinsic sinus rate of the dog (Evans et al., 1990; Du et al., 2017) and human (Opthof, 2000; Li et al., 2017) are similar, the higher parasympathetic tone in the dog is associated with a more pronounced sinus arrhythmia that we hypothesize based on the identified patterning of intervals in this study is, in part, the consequence of a more profound effect on the SACP resulting in exit block. Moreover, although variations in the non-linear patterns of sinus rhythm were seen in the dog, those with greater clustering of beats with ‘shorter’ short–long intervals had less variability than those with greater branching suggesting a possible difference in the balance between the rhythmicity of the parasympathetic and sympathetic tone. Of course, further studies are require to confirm these hypotheses.

### SACP and Bifurcation Interval

Detailed and expansive studies of the canine sinus node support the existence of SACP that are subject to exit block during certain perturbations that mimic parasympathetic modulation. Moreover, the potential for decremental conduction would support modulation of exit block and this would then explain the inexact multiples and clustering of intervals. Experimental studies show that acetylcholine or adenosine can influence pacing cell depolarization and conduction through the SACP (Fedorov et al., 2009, 2010, 2012; Glukhov et al., 2013; Lou et al., 2013, 2014). Therefore, it is reasonable to hypothesize that our results, which demonstrate clustered and branched patterns related to the ratio of short- and long- intervals in the dog, could follow the same modulation of rate and rhythm via exit block through the SACP and slowed phase four-depolarization of pacing cells. In dogs with the longer beat-to-beat intervals a ‘cloud’ widely surrounding the line of identity may indicate a more profound effect of impulse initiation rather than conduction out the SACP. This type of pattern was identified in all dogs administered hydromorphone which is known to have a parasympathomimetic effect on the sinus node. This observation maybe concordant with saturation of the parasympathetic effect on the sinus node decreasing the respiratory modulation of heart rate variability. The latter is identified in trained athletes (Goldberger et al., 1994). We corrected for the mathematical biased inherent in time and frequency analyses by dividing by the standard deviation or standard deviation squared, respectively (Sacha and Pluta, 2008; Billman, 2011, 2013; Billman et al., 2015b; Sacha et al., 2013). Finally, it is intriguing that the bifurcation interval at which point slowing of the heart rate in the dog becomes non-linear, a very narrow 95% confidence range of 97.8–102.8 beats per minute (583.5–613.5 ms) approximates the intrinsic sinus node rate of the adult and older dog. The intrinsic rate of the sinus node is that which is inherent to spontaneous depolarization of the sinus node cells without autonomic input (Billman et al., 2015a). How autonomic input is subtracted (e.g., pharmacological blockade, surgical denervation, explanted heart) influences this value (Evans et al., 1990). Also, age is an important determinant of the intrinsic rate with young dogs (168 ± 11 bpm) having faster rates compared to adults (120 ± 9 beats per minute) and elderly (88 ± 9 bpm) dogs (Du et al., 2017).

### Heart Rate Variability

Heart rate variability is influenced by multiple inputs of central and peripheral parasympathetic and sympathetic modulation (Evans et al., 1990; Goldberger et al., 1994; Cerutti et al., 1995; Roossien et al., 1997; Stein et al., 2005; Billman, 2011, 2013; Billman et al., 2015b; Shaffer and Ginsberg, 2017; Behar et al., 2018a, b). The variability results from complex interactions and cannot be simplified to say that high variability universally indicates high parasympathetic modulation (Goldberger et al., 1994; Costa et al., 2017; Shaffer and Ginsberg, 2017; Hayano and Yuda, 2019). Both groups of dogs in this study had greater variability using traditional time domain indices than in humans. Traditional methods to evaluate the variability of the sinus node driven rate in humans have meaningful limitations particularly when evaluating disease states and this has prompted advanced methods (Hayano and Yuda, 2019). Some indices used in the evaluation of the rhythm in humans are not applicable to the dog. For example, the percentage of successive R–R intervals that differ by more than 50 ms (pNN50) because this difference is too small for the dog. The triangular index is not applicable because the dog does not have a singular Gaussian distribution (Shaffer and Ginsberg, 2017). Furthermore, some of the linear measurements used in the evaluation of Poincaré plots in humans are also not valid in the dog because they are derived from the studies of linear changes along the line of identity (Shaffer and Ginsberg, 2017). These include the area of the ellipse (width/length), which represents total heart rate variability, the standard deviation perpendicular to the line of identity and the standard deviation along the line of identity. Newly developed methods to better understand the variability in the sinus rhythm have been developed and deserve further evaluation in different species (Costa et al., 2017).

### Beyond Visual Geometric Analyses

Although this study used dynamic Poincaré plots and three-dimensional imaging to demonstrate a difference in beat-to-beat intervals between the dog and human these visual indices are inadequate (Esperer et al., 2008). Moreover, linear quantification are inappropriate, thus non-linear analyses demand exploration. These may include approximate or sample entropy, detrended fluctuation analyses and fractal measures, as well as the development of new methodologies through computer modeling of the beat-to-beat variation (Esperer et al., 2008; Nicolini et al., 2012; Khandoker et al., 2013; Burykin et al., 2014; Yaniv et al., 2014b; Henriques et al., 2015; Shaffer and Ginsberg, 2017; Borracci et al., 2018; Valente et al., 2018). In humans, slowing of the heart rate is a continuum with some having slower rates (e.g., athletes), but in the normal dog slowing of the heart is not a continuous process. We hypothesize that the dog has greater potential for alterations of conduction in the SACP that is linked to the parasympathetic modulation. This hypothesis is supported by the (1) abrupt change of beat-to-beat intervals, (2) paucity and grouping of beats rather than a continuum of beat intervals, and (3) relatively consistent bifurcation interval during basal conditions.

### Clinical Relevance

Both humans and dogs can be afflicted with sinus node dysfunction. Such dysfunction may be intrinsic, extrinsic or both. Alterations in the parasympathetic nervous system or molecular targets likely play an important role, thus understanding the relationship of the sinus node complex with the inclusion of the SACP is likely vital to differentiating disease that results in exit block versus those with disease from impulse formation. The use of the techniques illustrated herein may be valuable in this differentiation.

### Limitations

Our study is limited to the surface electrocardiogram without direct assessment of the multiplicity of inputs that control the sinus rhythm. However, the patterning observed supports experimental studies of the sinus node in the dog. The study population of dogs and humans included a wide range of ages; however, the range in age between boxers and non-boxers was different and likely responsible for the difference in the relationship of age to time domain indices of heart rate variability. Equating dog age to human age is known to be difficult, non-linear and highly variable depending on the breed of dog (Cotman and Head, 2008). Consequently, attempts to compare the influence of age between the dog and human from our study is not possible. It is known that sinus node function varies with age, and, thus, this must be taken into consideration. Additionally, because 32 different breeds were studied of varied sizes with 108 dogs neutered, comparison to humans by weight or sex was not undertaken. Although the only entry criteria that differed between the dogs and humans was the duration that defined a sinus pause, this did not impact the difference in beat-to-beat patterning because the non-linear change occurred at intervals that were more than 1000 ms shorter than the defined pause. It is stressed that autonomic activity was not directly measured, and heart rate variability provides only an indirect qualitative assessment of cardiac parasympathetic activity. More advanced analyses need to be undertaken to further investigate the potential mechanisms for the unique patterning of sinus arrhythmia in the dog.

## Conclusion

Our study demonstrated distinctive differences in beat-to-beat sinus rhythms in the dog compared to humans. Specific patterns within and between dog were associated with differences in heart rate, time and frequency domain variability. Treatment with atropine as a parasympatholytic agent resulted in small variation in beat intervals that were along the line of identity while treatment with hydromorphone as a parasympathomimetic agent resulted in an expansion and exaggeration of the patterns identified without linear variation in the beat-to-beat intervals. The non-linear rhythms of sinus arrhythmia in the dog require assessment using analyses appropriate to the dynamics. The multiplicity of input that results in the beat-to-beat dynamics identified in the normal canine are concordant with the possibility of not only alterations in the initiation of sinus impulses, but also variable exit block within the SACP. Furthermore, these results may offer insight to the possible mechanisms for sinus node dysfunction seen in the dog that has a disease footprint similar to humans.

## Data Availability Statement

The datasets generated for this study are available on request to the corresponding author.

## Ethics Statement

The animal study was reviewed and approved by the Cornell University IACUC for drug studies. Written informed consent was obtained from the owners for the participation of their animals in this study.

## Author Contributions

NM designed the study, analyzed the data, created all the images, interpreted the data, and wrote the manuscript. WF created the software for the analysis and contributed to the interpretation of the data. RP contributed to the interpretation of the data and reviewed the manuscript.

## Conflict of Interest

The authors declare that the research was conducted in the absence of any commercial or financial relationships that could be construed as a potential conflict of interest.

## Footnotes

1 http://wyattflanders.com/poincare/

2 http://wyattflanders.com/poincareplot/

## Abbreviations

RMSSD: square root of the mean squared successive interval differences
R–R intervals or beat-to-beat intervals: duration between two QRS complexes (R–R intervals or beat-to-beat intervals) and verified surrogate for P–P intervals for this study
SACP: sinoatrial conduction pathways
SDANN: standard deviation of all 5-min beat interval means
SDNN: Standard deviation of all beat intervals
SDNNIn: mean of all 5-min beat interval standard deviations (index)
