# Supplementary material for: Beat-to-Beat Patterning of Sinus Rhythm Reveals Non-linear Rhythm in the Dog Compared to the Human
Source: Front Physiol. 2020 Jan 22;10:1548. doi: 10.3389/fphys.2019.01548 (PMC6990411; doi:10.3389/fphys.2019.01548)
Supplement: Supplementary file 1 [file Data_Sheet_1.zip › Supplementary Material/Suplementary Table 1.docx]

**Supplementary Table 1**. Relationship of age with heart rate and 24-hour time domain heart rate variability parameters between boxers, non-boxers and humans. Heart rate variability parameters corrected for heart rate (see text). The *p* values are in parentheses and corrected for the 5 parameters tested with Bonferroni correction. *Data shown are slope and standard error.

| **Parameter** | **Boxer (n = 69 )** | **Non-boxer (n =61)** | **Human (n = 40)** |
| --- | --- | --- | --- |
| Heart rate (bpm) | 0.1968 +/- 0.4350  (1.0) | 1.522 +/- 0.4113  (.034) | -0.0824 +/- 0.0988  (1.0) |
| SDNN (ms) | -0.0042 +/- 0.0023  (.35) | -0.0052 +/- 0.0027  (.27) | -0.0013 +/- 0.0004  (0.015) |
| SDANNIn (ms) | -0.003 +/- 0.0022  (.95) | -0.0034 +/- 0.0023  (1.0) | -0.0008 +/- 0.0002  (.0005) |
| SDANN (ms) | -0.0034 +/- 0.0019  (.35) | -0.0055 +/- 0.0022  (.08) | -0.0012 +/- 0.0004  (.04) |
| RMSSD (ms) | -0.0045 +/- 0.0031  (.8) | -0.0008 +/- 0.0046  (1.0) | -0.0006 +/- 0.0002  (.018) |
