# Supplementary material for: Beat-to-Beat Patterning of Sinus Rhythm Reveals Non-linear Rhythm in the Dog Compared to the Human
Source: Front Physiol. 2020 Jan 22;10:1548. doi: 10.3389/fphys.2019.01548 (PMC6990411; doi:10.3389/fphys.2019.01548)
Supplement: Supplementary file 1 [file Data_Sheet_1.zip › Supplementary Material/Supplementary Video 1 and 2.pptx]

## Slide 1
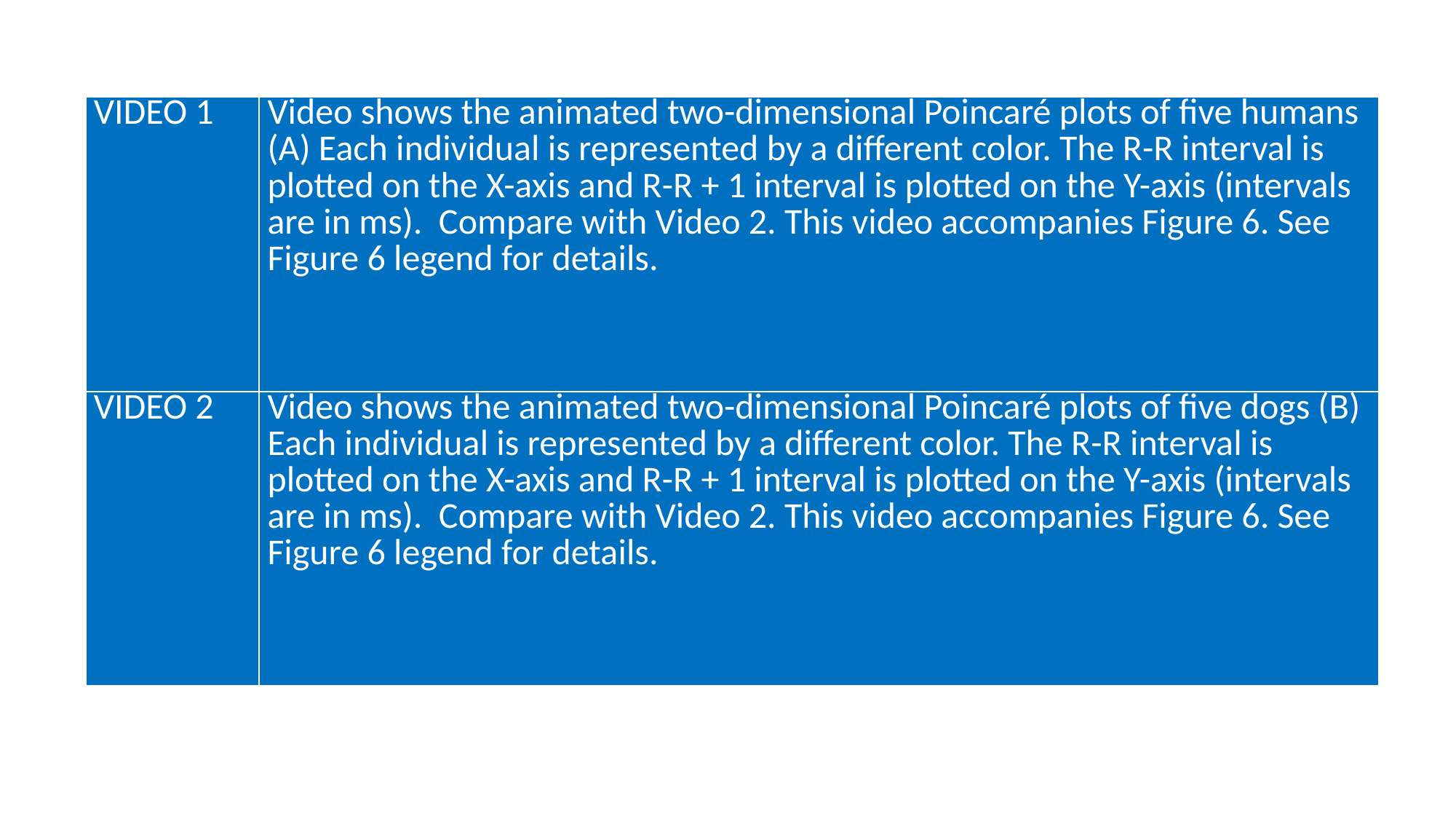

| VIDEO 1 | Video shows the animated two-dimensional Poincaré plots of five humans (A) Each individual is represented by a different color. The R-R interval is plotted on the X-axis and R-R + 1 interval is plotted on the Y-axis (intervals are in ms). Compare with Video 2. This video accompanies Figure 6. See Figure 6 legend for details. |
| --- | --- |
| VIDEO 2 | Video shows the animated two-dimensional Poincaré plots of five dogs (B) Each individual is represented by a different color. The R-R interval is plotted on the X-axis and R-R + 1 interval is plotted on the Y-axis (intervals are in ms). Compare with Video 2. This video accompanies Figure 6. See Figure 6 legend for details. |

## Slide 2
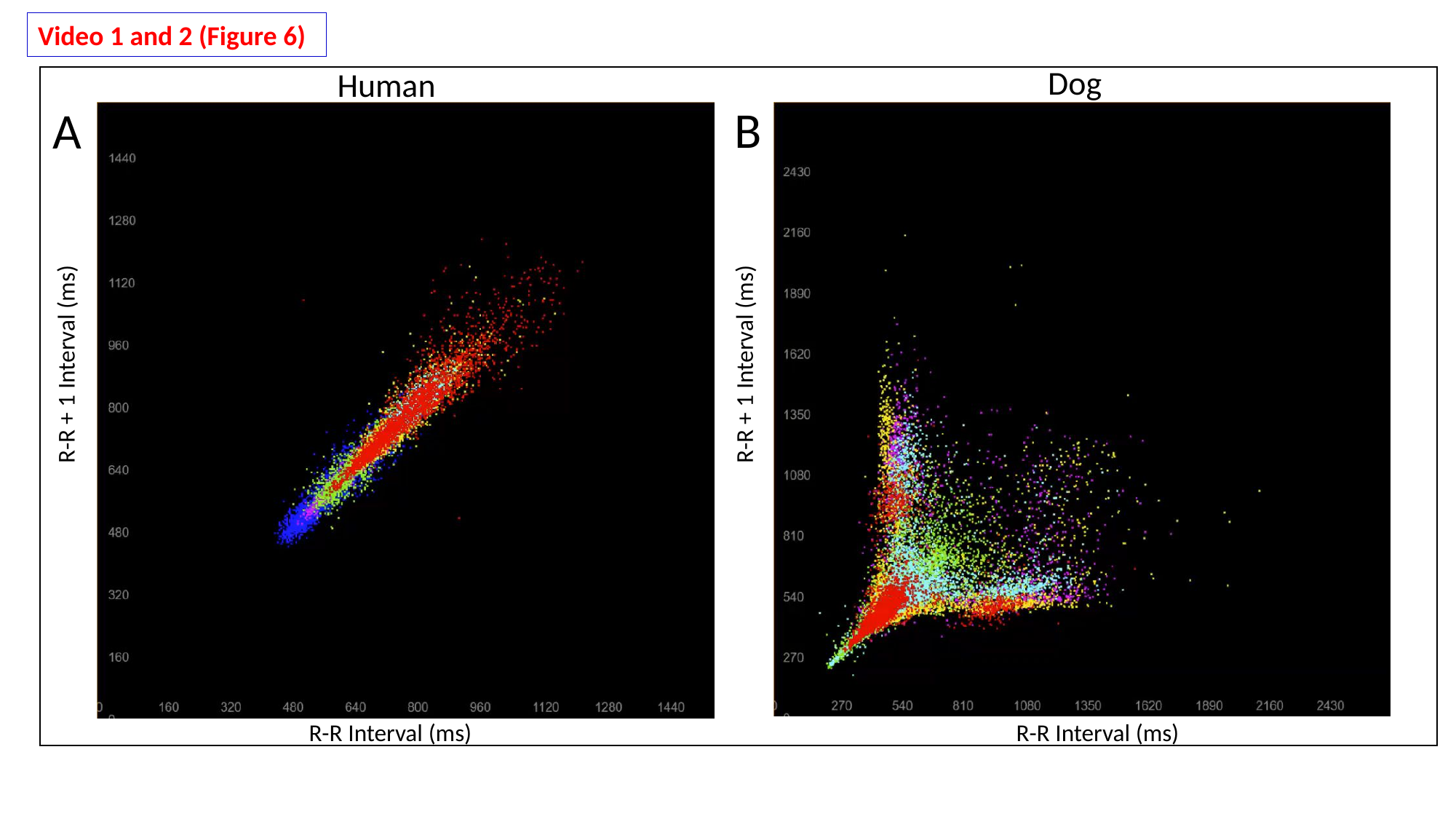

Video 1 and 2 (Figure 6)
Dog
Human
B
A
R-R + 1 Interval (ms)
R-R + 1 Interval (ms)
R-R Interval (ms)
R-R Interval (ms)
