# Supplementary material for: Beat-to-Beat Patterning of Sinus Rhythm Reveals Non-linear Rhythm in the Dog Compared to the Human
Source: Front Physiol. 2020 Jan 22;10:1548. doi: 10.3389/fphys.2019.01548 (PMC6990411; doi:10.3389/fphys.2019.01548)
Supplement: Supplementary file 1 [file Data_Sheet_1.zip › Supplementary Material/Supplementary Video 3.pptx]

## Slide 1
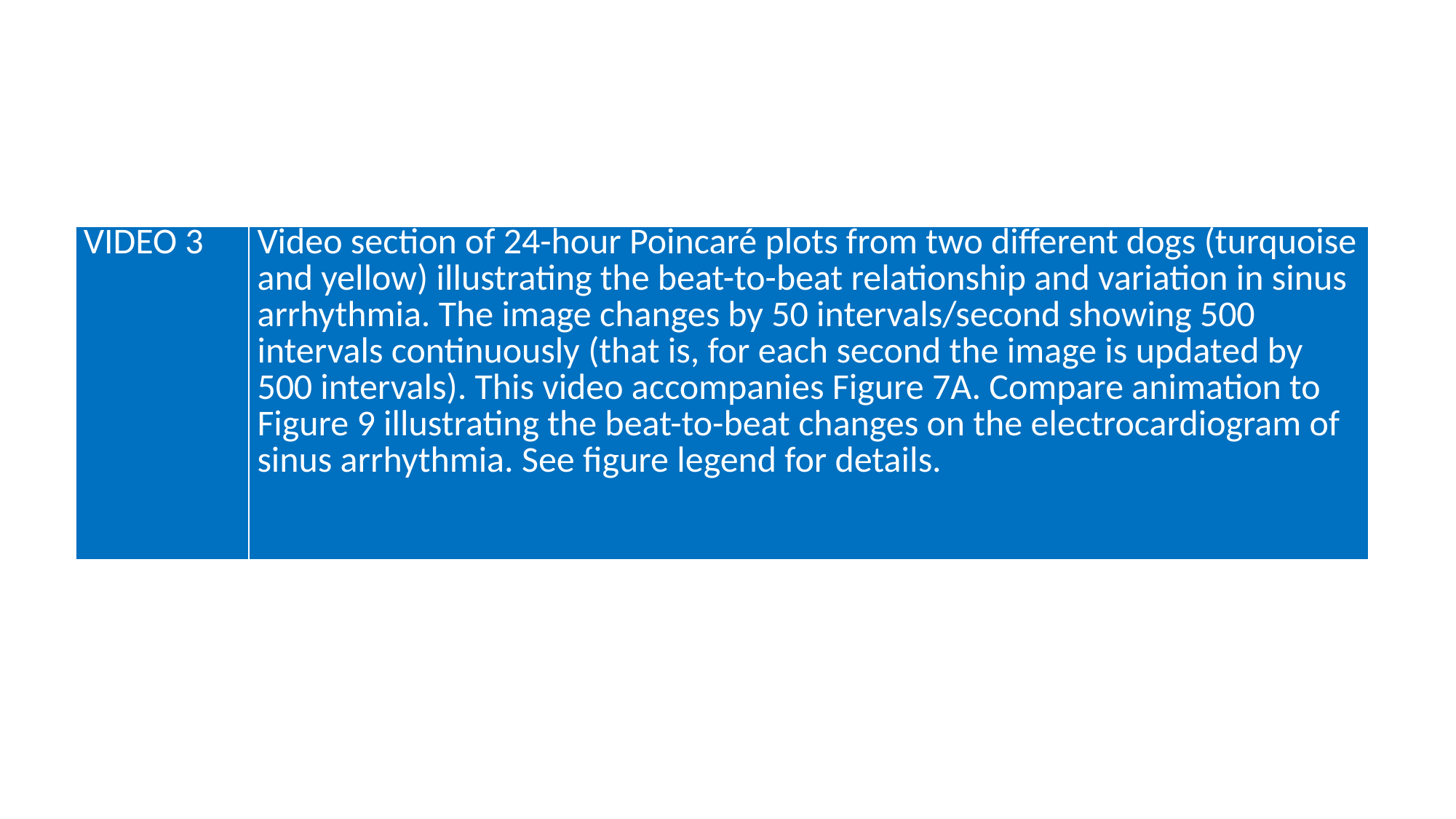

| VIDEO 3 | Video section of 24-hour Poincaré plots from two different dogs (turquoise and yellow) illustrating the beat-to-beat relationship and variation in sinus arrhythmia. The image changes by 50 intervals/second showing 500 intervals continuously (that is, for each second the image is updated by 500 intervals). This video accompanies Figure 7A. Compare animation to Figure 9 illustrating the beat-to-beat changes on the electrocardiogram of sinus arrhythmia. See figure legend for details. |
| --- | --- |

## Slide 2
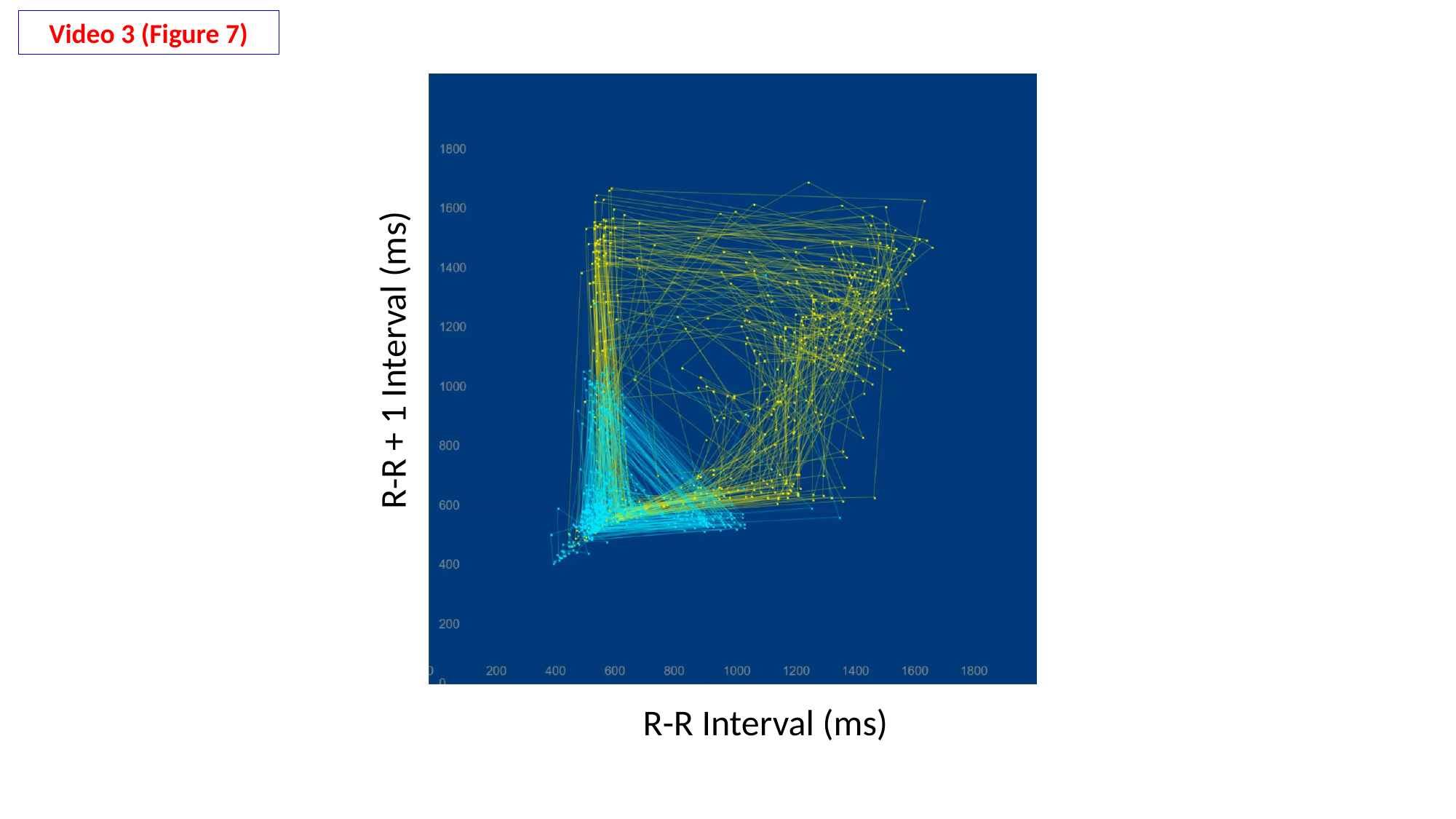

Video 3 (Figure 7)
R-R + 1 Interval (ms)
R-R Interval (ms)
