# Supplementary material for: Beat-to-Beat Patterning of Sinus Rhythm Reveals Non-linear Rhythm in the Dog Compared to the Human
Source: Front Physiol. 2020 Jan 22;10:1548. doi: 10.3389/fphys.2019.01548 (PMC6990411; doi:10.3389/fphys.2019.01548)
Supplement: Supplementary file 1 [file Data_Sheet_1.zip › Supplementary Material/Supplementary Video 5.pptx]

## Slide 1
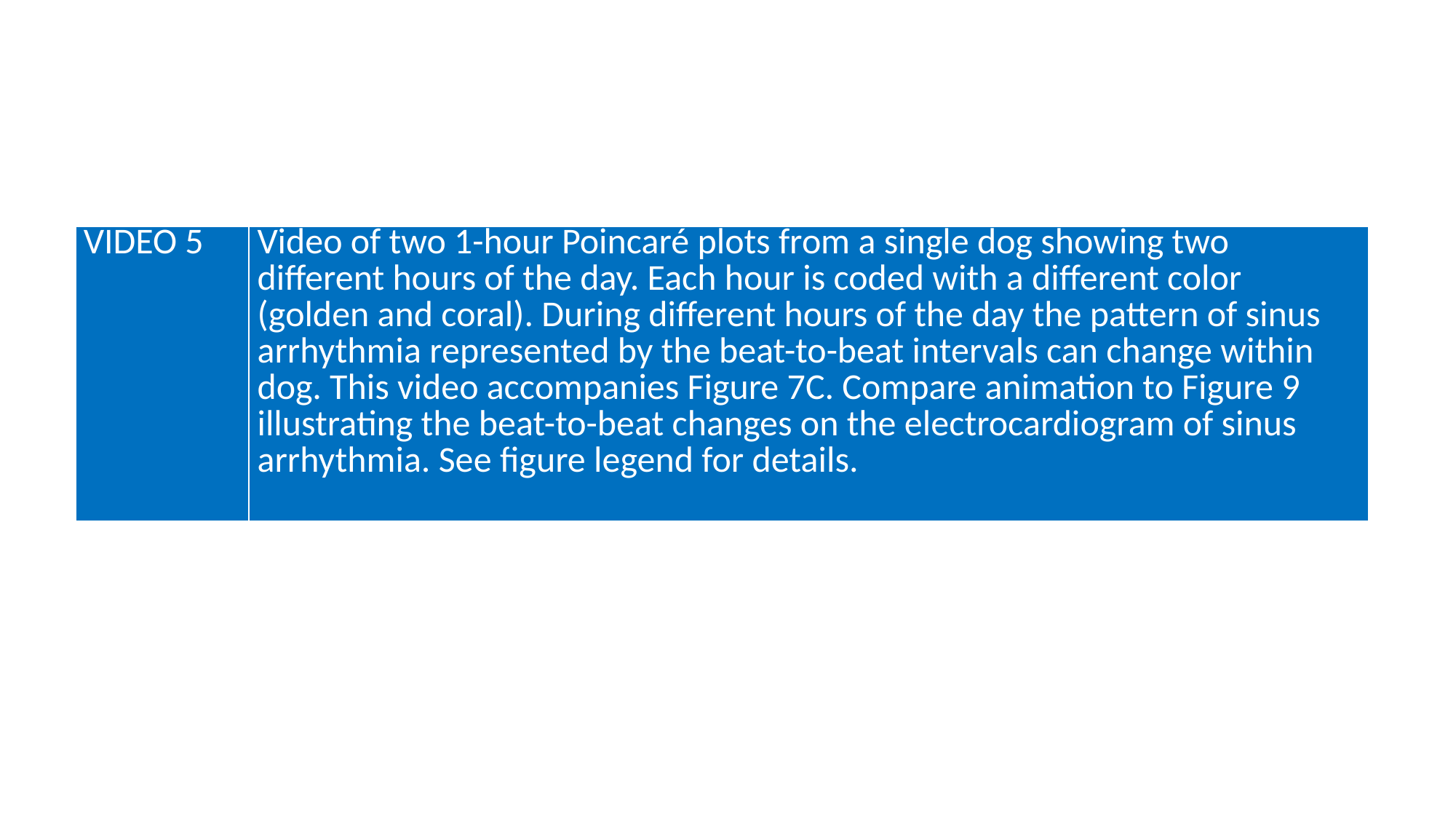

| VIDEO 5 | Video of two 1-hour Poincaré plots from a single dog showing two different hours of the day. Each hour is coded with a different color (golden and coral). During different hours of the day the pattern of sinus arrhythmia represented by the beat-to-beat intervals can change within dog. This video accompanies Figure 7C. Compare animation to Figure 9 illustrating the beat-to-beat changes on the electrocardiogram of sinus arrhythmia. See figure legend for details. |
| --- | --- |

## Slide 2
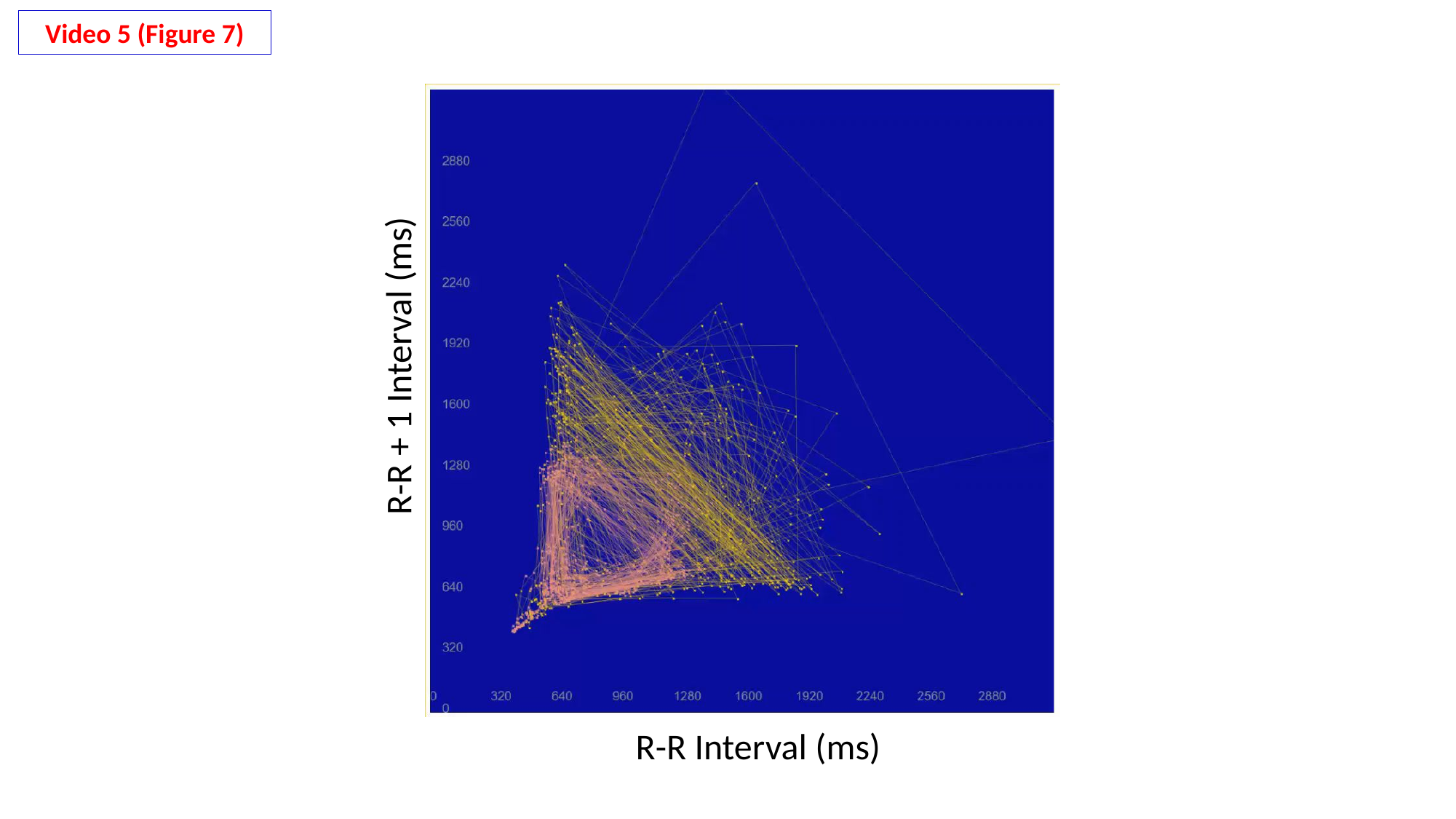

Video 5 (Figure 7)
R-R + 1 Interval (ms)
R-R Interval (ms)
