# Supplementary material for: Beat-to-Beat Patterning of Sinus Rhythm Reveals Non-linear Rhythm in the Dog Compared to the Human
Source: Front Physiol. 2020 Jan 22;10:1548. doi: 10.3389/fphys.2019.01548 (PMC6990411; doi:10.3389/fphys.2019.01548)
Supplement: Supplementary file 1 [file Data_Sheet_1.zip › Supplementary Material/Supplementary Video 7.pptx]

## Slide 1
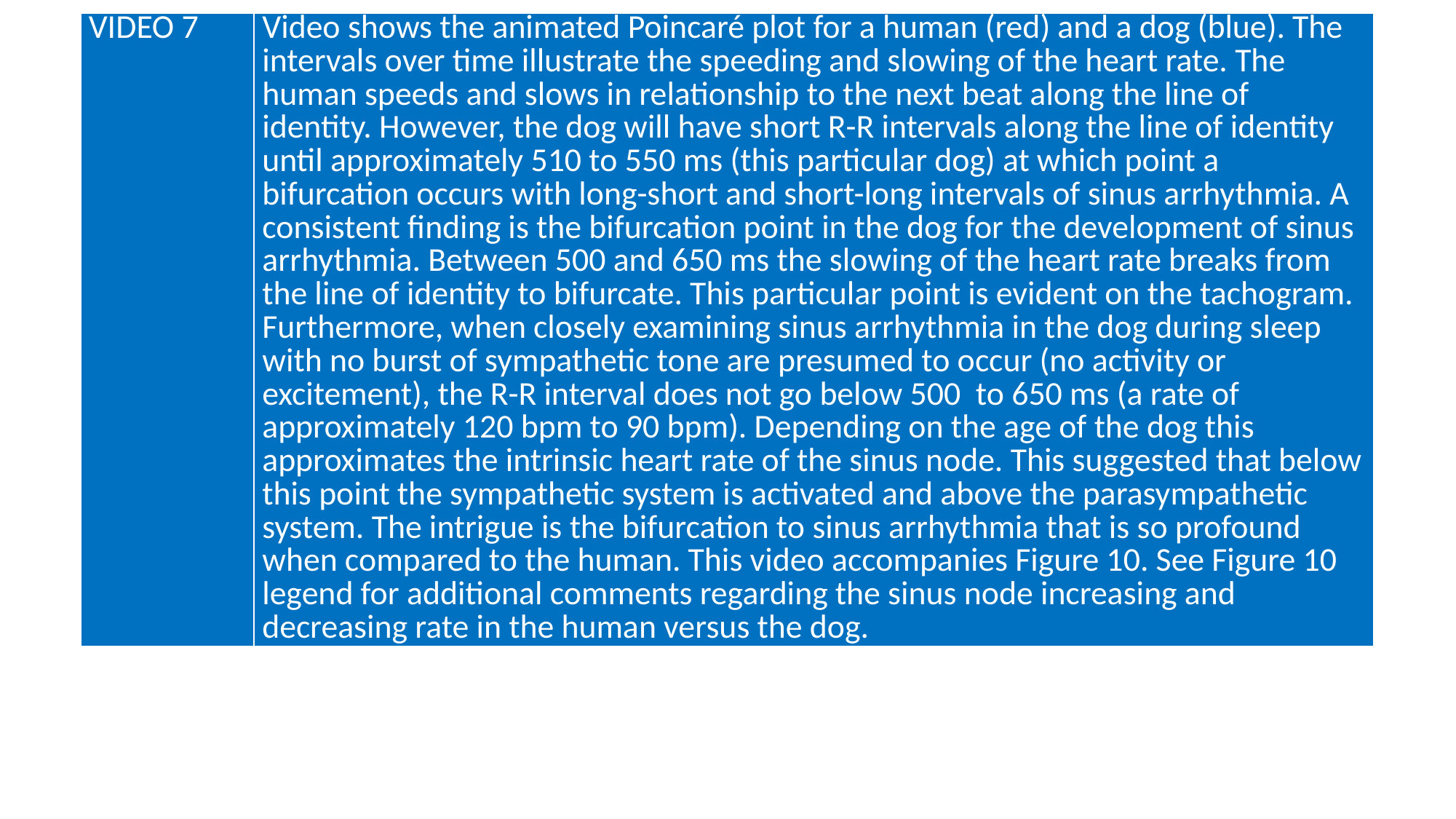

| VIDEO 7 | Video shows the animated Poincaré plot for a human (red) and a dog (blue). The intervals over time illustrate the speeding and slowing of the heart rate. The human speeds and slows in relationship to the next beat along the line of identity. However, the dog will have short R-R intervals along the line of identity until approximately 510 to 550 ms (this particular dog) at which point a bifurcation occurs with long-short and short-long intervals of sinus arrhythmia. A consistent finding is the bifurcation point in the dog for the development of sinus arrhythmia. Between 500 and 650 ms the slowing of the heart rate breaks from the line of identity to bifurcate. This particular point is evident on the tachogram. Furthermore, when closely examining sinus arrhythmia in the dog during sleep with no burst of sympathetic tone are presumed to occur (no activity or excitement), the R-R interval does not go below 500 to 650 ms (a rate of approximately 120 bpm to 90 bpm). Depending on the age of the dog this approximates the intrinsic heart rate of the sinus node. This suggested that below this point the sympathetic system is activated and above the parasympathetic system. The intrigue is the bifurcation to sinus arrhythmia that is so profound when compared to the human. This video accompanies Figure 10. See Figure 10 legend for additional comments regarding the sinus node increasing and decreasing rate in the human versus the dog. |
| --- | --- |

## Slide 2
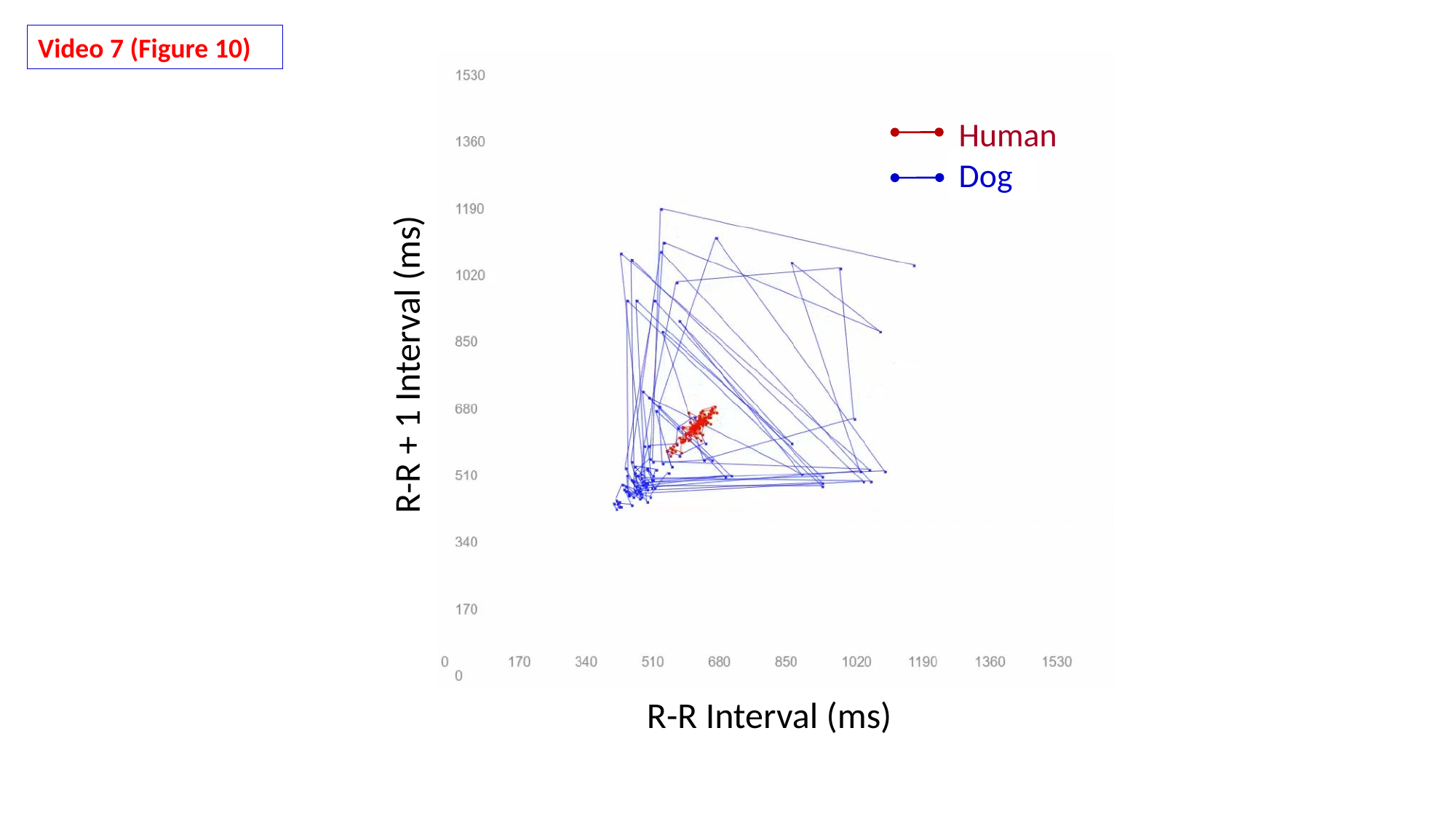

Video 7 (Figure 10)
Human
Dog
R-R + 1 Interval (ms)
R-R Interval (ms)
