# Supplementary material for: Beat-to-Beat Patterning of Sinus Rhythm Reveals Non-linear Rhythm in the Dog Compared to the Human
Source: Front Physiol. 2020 Jan 22;10:1548. doi: 10.3389/fphys.2019.01548 (PMC6990411; doi:10.3389/fphys.2019.01548)
Supplement: Supplementary file 1 [file Data_Sheet_1.zip › Supplementary Material/Supplementary Video 8.pptx]

## Slide 1
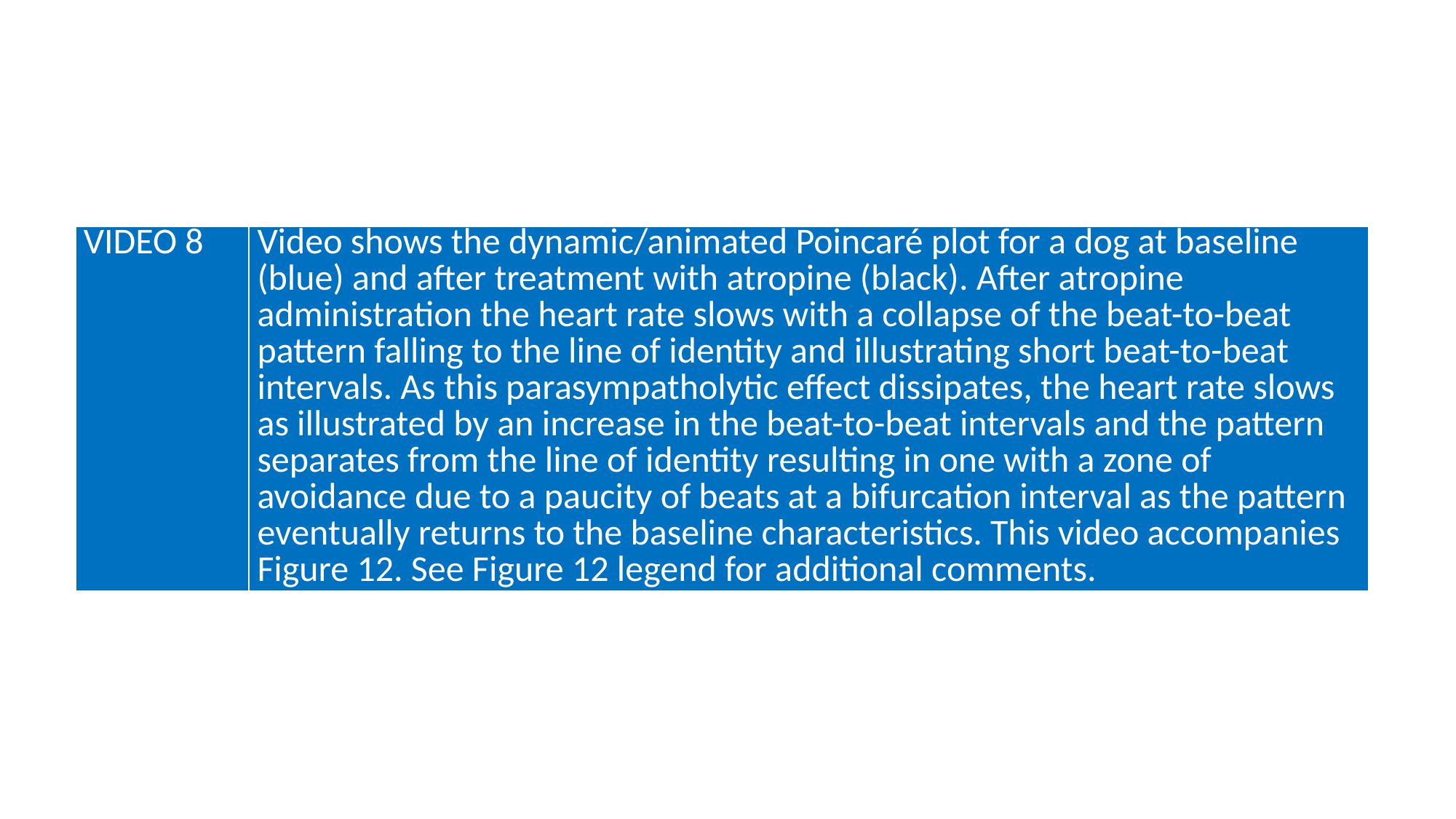

| VIDEO 8 | Video shows the dynamic/animated Poincaré plot for a dog at baseline (blue) and after treatment with atropine (black). After atropine administration the heart rate slows with a collapse of the beat-to-beat pattern falling to the line of identity and illustrating short beat-to-beat intervals. As this parasympatholytic effect dissipates, the heart rate slows as illustrated by an increase in the beat-to-beat intervals and the pattern separates from the line of identity resulting in one with a zone of avoidance due to a paucity of beats at a bifurcation interval as the pattern eventually returns to the baseline characteristics. This video accompanies Figure 12. See Figure 12 legend for additional comments. |
| --- | --- |

## Slide 2
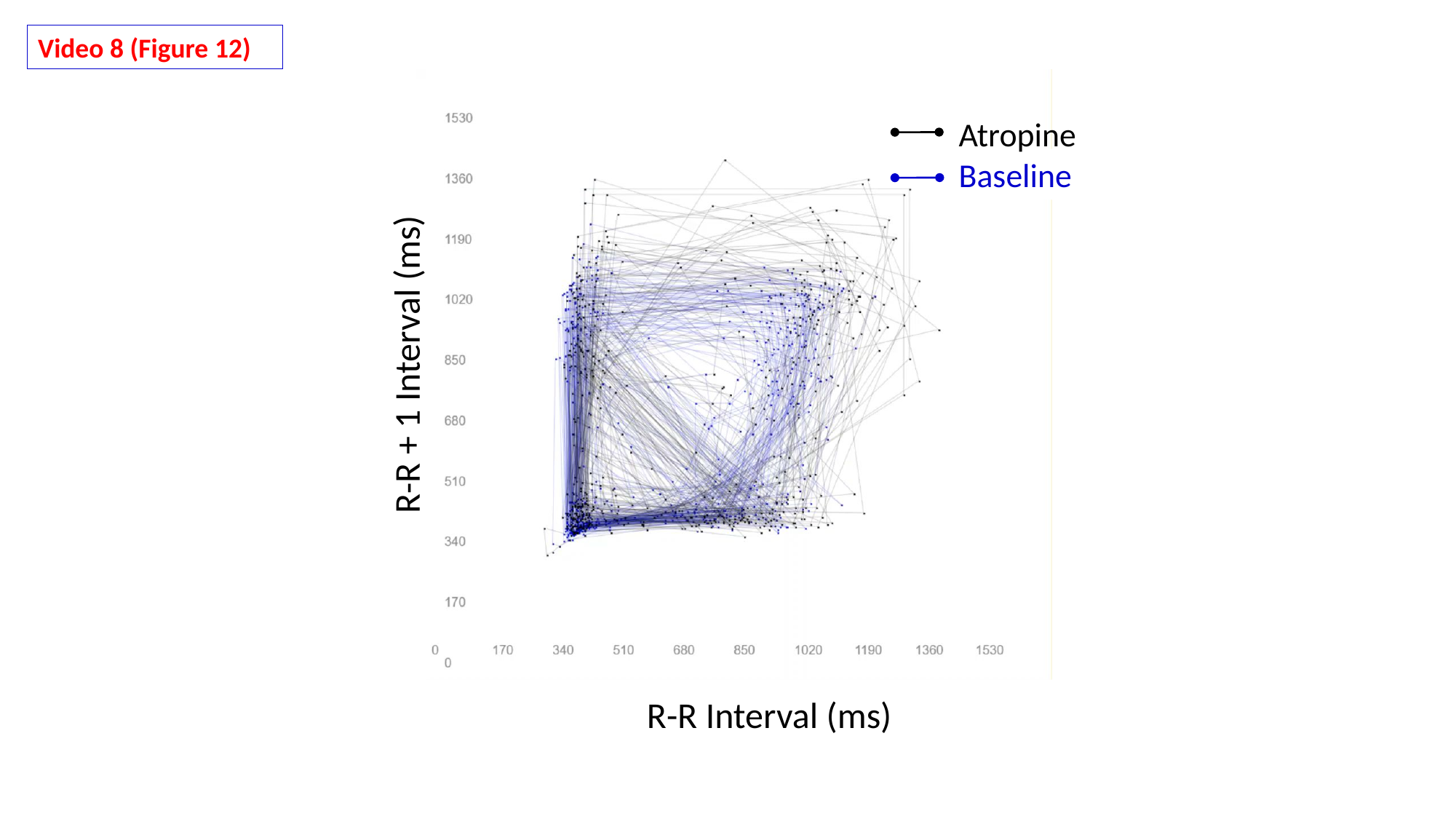

Video 8 (Figure 12)
Atropine
Baseline
R-R + 1 Interval (ms)
R-R Interval (ms)
