# Supplementary material for: Beat-to-Beat Patterning of Sinus Rhythm Reveals Non-linear Rhythm in the Dog Compared to the Human
Source: Front Physiol. 2020 Jan 22;10:1548. doi: 10.3389/fphys.2019.01548 (PMC6990411; doi:10.3389/fphys.2019.01548)
Supplement: Supplementary file 1 [file Data_Sheet_1.zip › Supplementary Material/Supplementary Video 9.pptx]

## Slide 1
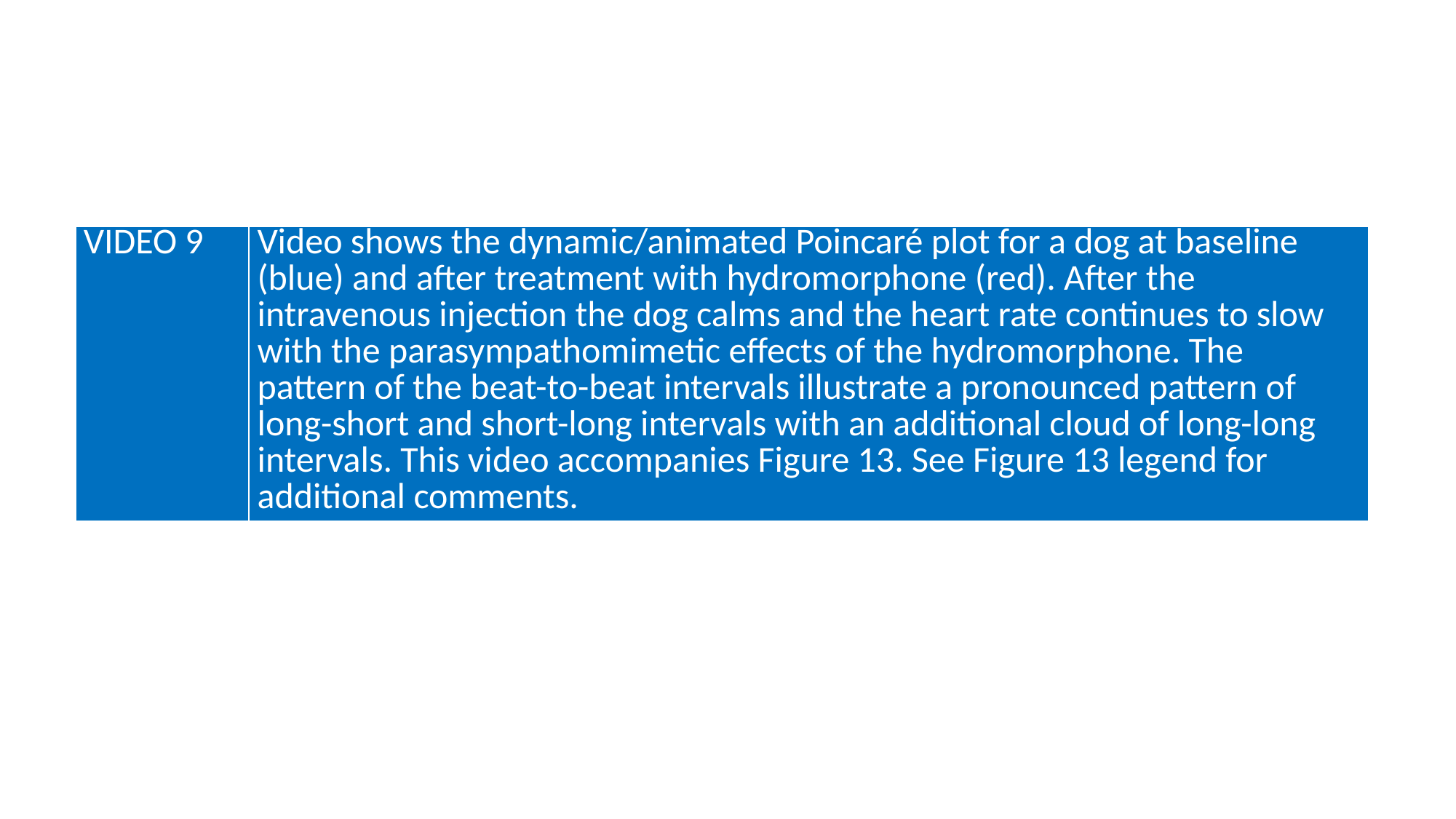

| VIDEO 9 | Video shows the dynamic/animated Poincaré plot for a dog at baseline (blue) and after treatment with hydromorphone (red). After the intravenous injection the dog calms and the heart rate continues to slow with the parasympathomimetic effects of the hydromorphone. The pattern of the beat-to-beat intervals illustrate a pronounced pattern of long-short and short-long intervals with an additional cloud of long-long intervals. This video accompanies Figure 13. See Figure 13 legend for additional comments. |
| --- | --- |

## Slide 2
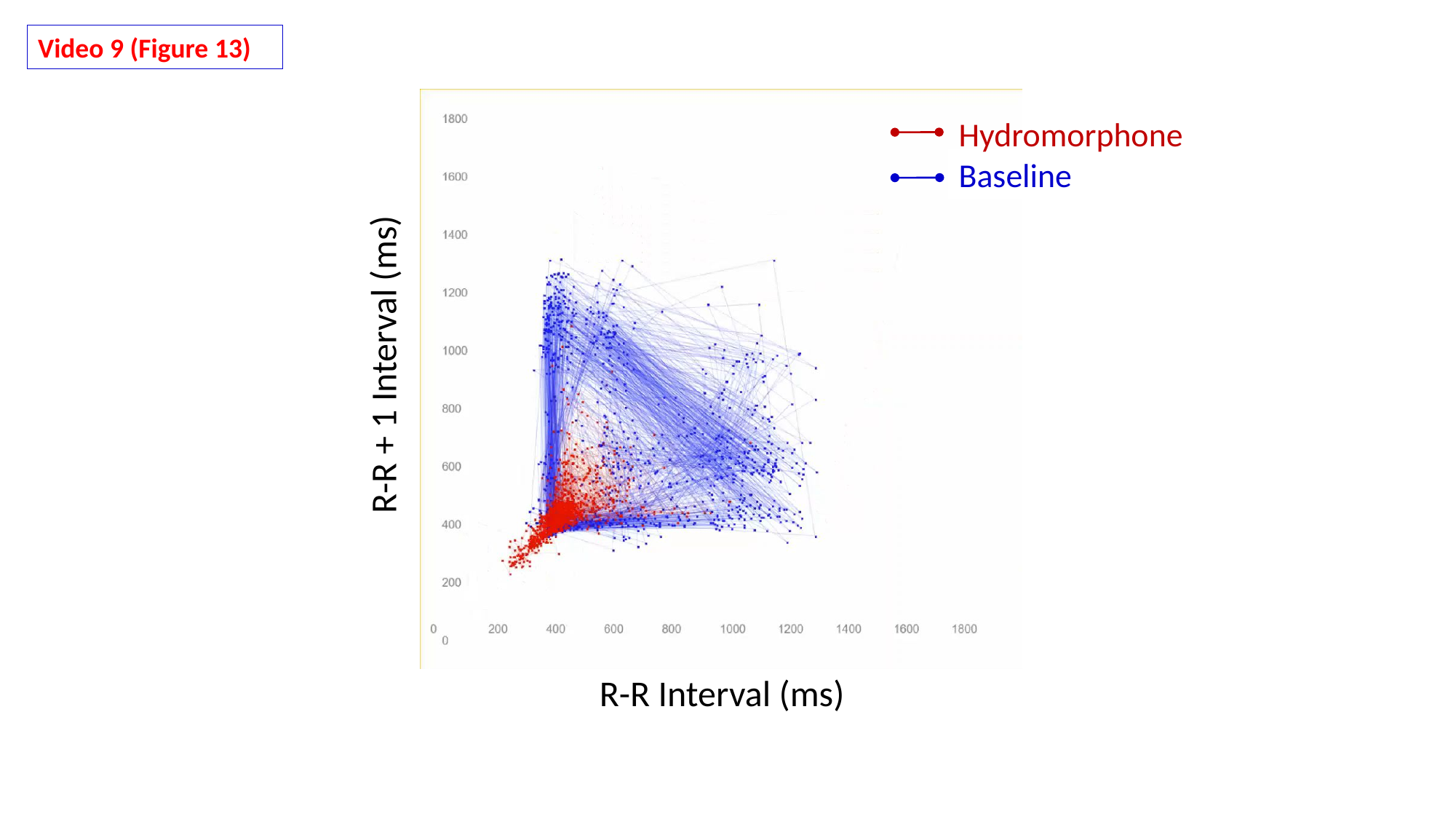

Video 9 (Figure 13)
Hydromorphone
Baseline
R-R + 1 Interval (ms)
R-R Interval (ms)
